# Effect of terahertz radiation on cells and cellular structures

**DOI:** 10.1007/s12200-024-00146-y

**Published:** 2025-01-27

**Authors:** A. P. Rytik, V. V. Tuchin

**Affiliations:** 1 Institute of Physics, Saratov State University, Saratov, 410012 Russia; 2 Science Medical Center, Saratov State University, Saratov, 410012 Russia; 3 Laboratory of Laser Molecular Imaging and Machine Learning, Tomsk State University, Tomsk, 634050 Russia; 4 Institute of Precision Mechanics and Control, Federal Research Center “Saratov Scientific Center of the Russian Academy of Sciences”, Saratov, 410012 Russia

**Keywords:** Electromagnetic radiation, Terahertz radiation, Biological effects, Living cell, Cancer cells

## Abstract

The paper presents the results of modern research on the effects of electromagnetic terahertz radiation in the frequency range 0.5–100 THz at different levels of power density and exposure time on the viability of normal and cancer cells. As an accompanying tool for monitoring the effect of radiation on biological cells and tissues, spectroscopic research methods in the terahertz frequency range are described, and attention is focused on the possibility of using the spectra of interstitial water as a marker of pathological processes. The problem of the safety of terahertz radiation for the human body from the point of view of its effect on the structures and systems of biological cells is also considered.

**Graphical Abstract:**

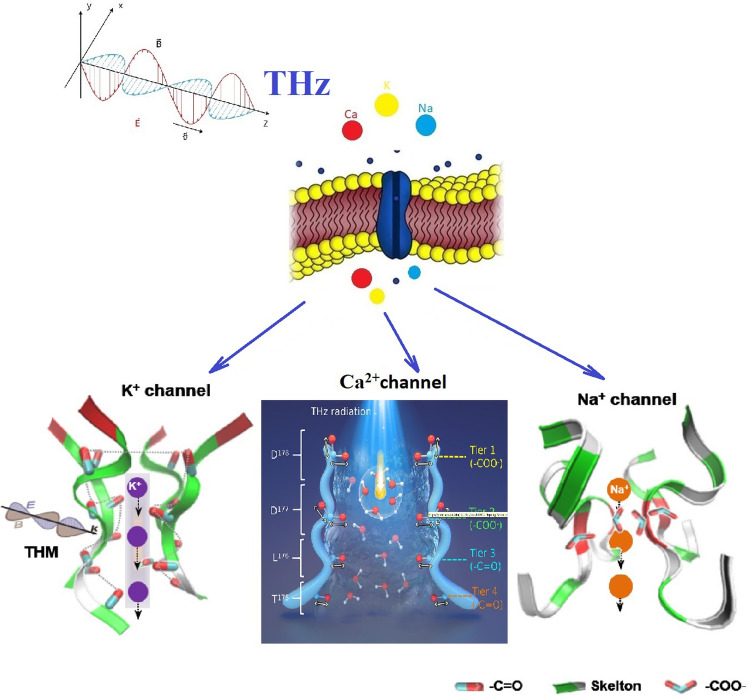

## Introduction

It is known that electromagnetic radiation (EMR) in the terahertz (THz) frequency range is well absorbed by a relatively thin layer of water, from 30 to 90% of which is contained in living tissues and cells. It is now a generally accepted scientific fact that THz radiation affects a biological cell and a living organism as a whole [1–4]. If we consider biological effects in and near the terahertz range, it is important to take into account frequencies from 0.1 to 100 THz [5–7]. This is primarily due to the various mechanisms of specific interaction of radiation with biological molecules as a result of radiation absorption with a change in the vibrational, rotational, or vibrational–rotational components of the motion of molecules [8–11]. This, in turn, can lead to local changes in the temperature of the cellular environment, ion channel activity and transmembrane capacitive charge. However, to study the biological effects that occur in the structures of a living cell under the action of THz radiation, the mechanisms of non-thermal action at low levels of power density and fluence are of great interest. Fundamentally, THz wave spectroscopy can make it possible to understand the processes occurring in living biological systems without changing, or admittedly little changing, the natural course of these processes. This is due to the low energy of THz photons compared to UV and visible radiation, the effect of which in the study of living objects can lead to breaks in covalent bonds and unacceptable changes in the structure of DNA and other biological molecules. Moreover, experimental techniques now make it possible to obtain high peak intensity of THz radiation (32 GW/cm^2^) for short pulse durations of the order of subpicoseconds, thereby minimizing the probability of thermal effects [12]. Ultimately, it turns out to be possible to study the non-thermal effects of THz radiation on fundamental processes of vital activity. At the same time, the use of modern THz radiation sources has advantages over traditional research methods based on laser spectroscopy. For example, a research group from Novosibirsk (Russia) uses the so-called Novosibirsk free electron laser (NovoFEL), which currently has the highest average and peak power in the world (wavelengths from 50 to 240 μm, peak power up to 1 MW) as a source of THz radiation for biological research [13]. This paper presents possible mechanisms of non-thermal effects of the response of *E. coli* cells to THz radiation. It has been shown that exposure to radiation with wavelengths of 130, 150 and 200 μm and average power density of 1.4 W/cm^2^ (pulse duration 50 ps and repetition rate 2.8–11.2 MHz) induces the expression of green fluorescent protein (GFP) in *E. coli* cells used as biosensors, which is recorded as a change in the intensity of their GFP fluorescence. The GFP expression depends on the radiation exposure and was observed for all studied wavelengths: for 5 min, it was not observed, for 10 min it was unstable, and after 15 min, GFP was always expressed. In all experiments, GFP was expressed for more than eight generations of cells after exposure to THz radiation. In other works of the same group [14–16], it was shown that THz radiation activates genes associated with the reaction to oxidative stress. The cells were irradiated at a wavelength of 130 μm with an intensity of 1.4 W/cm^2^ for 15 min with constant temperature control within 37 ± 2 °C. It has been shown that the effects of THz radiation are different for different stress systems of *E. coli* cells, in other words, THz radiation significantly affects oxidative homeostasis, which is vital for *E. coli* cells. The developed biosensors (promoters) are potentially applicable for the development of systems for monitoring the effects of THz radiation on the environment, biological cells and tissues, however, it is necessary to determine all the most likely mechanisms of THz influence on genetic changes in biological cells.

It has been shown that only weak Fröhlich condensates can be formed in a biological medium and, accordingly, have an influence on its chemical and enzymatic kinetics. A possible source of weak condensates may be the impact of a radio frequency, microwave or terahertz field on a biological object [11, 17, 18]. In several spectroscopic studies, such nonequilibrium movements of biological macromolecules have been modeled by large-scale vibrational modes. Theoretically, resonant frequencies in the range of 0.3–6.0 THz were found. Following Fröhlich’s theory, EMR in the THz range can excite such collective movements that could lead to structural changes in proteins, and, in turn, to shifts in chemical equilibrium. However, experimental studies at a relatively low power density of 0.8 mW/cm^2^ for bovine serum albumin (BSA) molecules and tubular protein complexes of tubulin, showed the absence of THz radiation effects in the similar frequency range 0.1–5.0 THz [18]. This may indicate that when exposed to such radiation, molecular effects are either below the detection limit of the measurement system itself, or the answer must be sought for other parameters of THz radiation.

These examples suggest that a systematic review of research on THz radiation action on biological cells and living organisms is needed. In particular, it is necessary to summaries existing studies of normal and cancer cells, namely the mechanisms of action, new spectroscopic data and therapeutic applications. Within the framework of the theranostic approach, when the same radiation with different energy parameters is used first for diagnostics (spectroscopy, imaging), and then with increased energy exposure for therapy, we can consider the use of THz radiation for the detection of various cancer cells presented in [19]. On the other hand, analysis of spectroscopic studies and especially terahertz imaging modalities must be carried out in the context of the radiation dose received by a living object during examination. The advantages and limitations of using THz for imaging in oncology are shown [19]:

The advantages include:

- Possibility of multiple scanning and monitoring of treatment, since the radiation is non-ionizing and therefore safe for a living organism. However, in connection with new works on the influence of THz radiation on the functioning of the genetic apparatus of a living cell, it is necessary to adjust the safety standards for the use of this radiation.
- Being very sensitive to polar molecules such as water, THz waves provide sufficiently high contrast images at the boundaries between healthy and tumor tissue, which can be a tool for the early detection of cancerous tumors and monitoring the treatment of cancer patients.
- The absence of strong Rayleigh scattering, due to the small size of cellular structures compared to the wavelength, makes it possible to increase the spatial resolution of images.
- Pathological disturbances accompanied by changes in tissue structure, the amount of free and bound water, ultimately affect the phase interaction of the wave with the tissue providing high phase sensitivity to the presence of water, the composition of tissue fluid and the dielectric constant of tissue.
- All types of biological tissues have characteristic absorption in the frequency range of the THz spectrum. However, not all of them are well studied at present.

Restrictions include:

Due to the large wavelength, the spatial resolution determined by the diffraction limit is comparatively small. However, using solid immersion microscopes, this drawback can be partially overcome [20–26].

- The penetration depth of THz waves into tissue is limited due to the strong absorption of interstitial water. However, here too there are options to overcome the limitation, for example, using pulsed irradiation [27, 28] or temporary and reversible tissue dehydration [29].
- Long scan times (due to spot imaging), low spatial resolution, and poor signal-to-noise ratio are limitations for THz imaging. These problems are partially overcome through the use of matrix THz radiation receivers. In particular, the Terasens device (Chernogolovka, Russia), consisting of a large multi-pixel array of terahertz radiation detectors [30].

It can be noted that most research in this area is focused on the detection of cancer tumors using THz radiation, while systematic studies of the mechanisms of the effects of radiation on normal and tumor cells are poorly represented in the literature.

This paper provides an overview of studies examining the effects of THz and near-THz radiation on normal and cancer cells, presents a systematization of the effects and reactions of biological systems based on a critical analysis of modern literature, discusses unresolved problems and puts forward new hypotheses.

## Interaction of terahertz radiation with cells and cellular structures

### Propagation and interaction of THz radiation with biological media

**Fig. 1:**
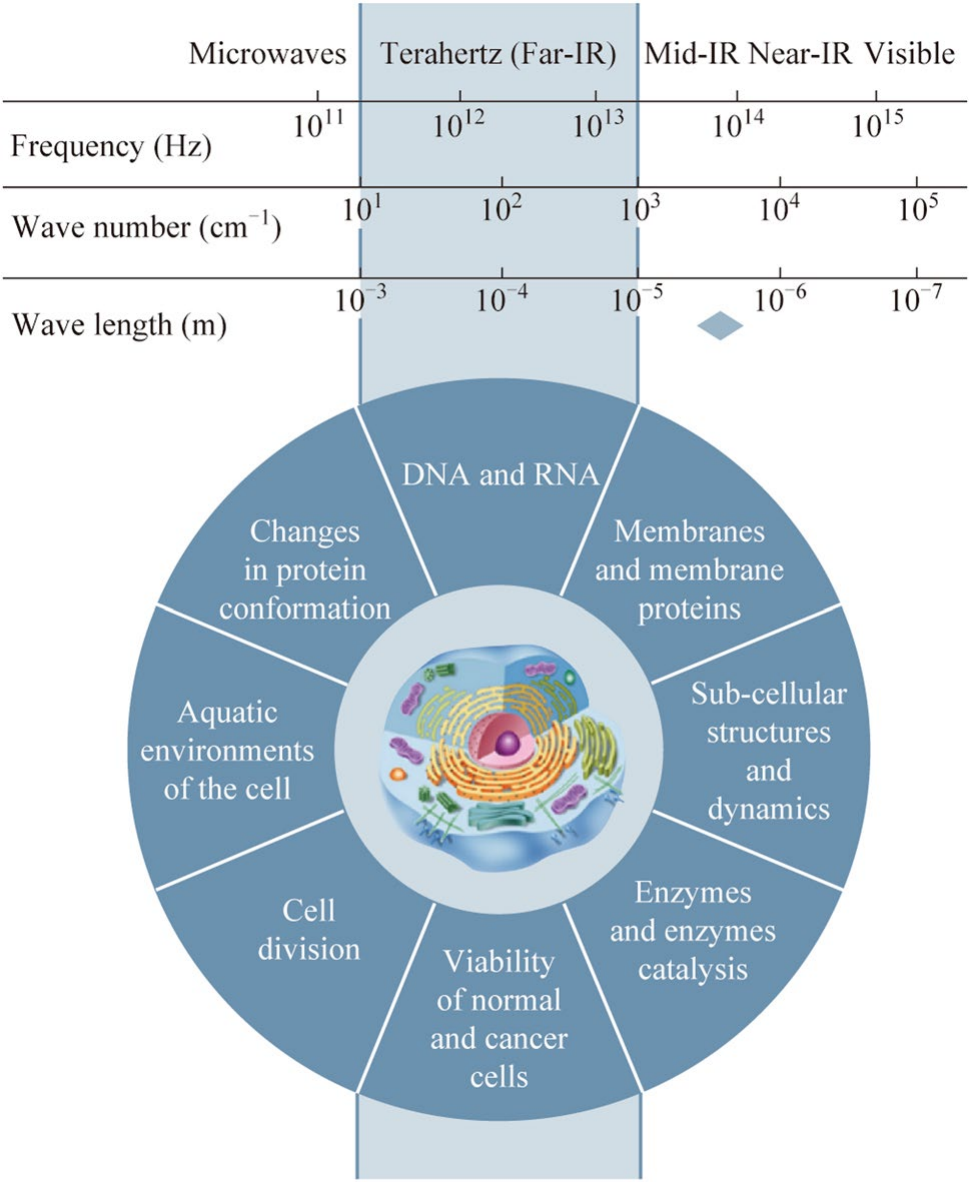
Identified various cellular targets for existing and future promising studies in the THz range, adapted from [32]

An analysis of the literature has shown that for fundamental research into the effects of THz radiation on biological objects, it is necessary to take into account the strong absorption of THz radiation by water, which is contained in large quantities in biological objects; temperature change with an accuracy of no worse than 0.01°; and evolutionary mechanisms of biological cell protection [31–33]. The temperature of the environment obviously needs to be controlled and stabilized if we are talking about fundamental research into non-thermal mechanisms of the influence of EMR. In addition, there are additional factors that need to be taken into account when designing studies, such as biological effects due to gene expression. Thus, in the review [34], it is noted that THz radiation can lead to abnormal gene expression, which depends on the experimental conditions. In general, a living cell can be represented as some biological object with targets for interaction with THz radiation and a corresponding response to such an effect, which is used or could potentially be used in biology and medicine (Fig. 1).

The important role of strong absorption of THz radiation by intra- and intercellular water is widely discussed, which is due to the non-trivial interaction of water molecules with biological molecules. Water is conventionally divided into bulk water (the molecules of which do not form bonds with biomolecules) and hydrated or bound water (it surrounds biomolecules and interacts with them) [35–37]. In general, the electromagnetic spectrum of a biological environment consists of three components: (1) Molecules of a dissolved substance (they do not experience strong relaxation, accompanied by a sharp increase in dielectric constant at low frequencies); they simply displace part of the volume of water; (2) Bound water, forming a hydrated shell around each solute molecule; (3) The remaining free water with the initial parameters—its contribution to the THz spectrum of the solution dominates even at a high concentration of the solution. Table 1 presents the effects characteristic for normal and malignant cellular structures and systems exposed to THz radiation, and Table 2 summarizes the data on the directions of cancer cell diagnostics using THz radiation, which is important for the development of theranostic technologies, where THz radiation from a single source is used first for diagnostics and then at increased energy parameters for therapy within a single protocol.

**Table 1:** Effects of THz radiation on normal and malignant cellular structures and systems

Cells, cellular structures or systems	Frequency (THz)	Field strength or intensity	Exposure time	Effects	References
Aquatic environments	1.39, 4.66, 10.86, 17.46, and 21.28	Up to 2.5 V/nm	−	Through molecular dynamics simulations, the authors proposed a phase transition to super permeation (approximately 1 order of magnitude enhancement) of confined water across a 1-D water channel caused by a THz electromagnetic stimulus with a limited thermal effect. The underlying mechanism is revealed to be a combination of strength matching and frequency resonance between a relatively weak stimulus and the hydrogen bond network of 1-D confined water, rather than the bulk water outside. This combination causes an anomalously structural phase transition of only the confined water while efficiently limiting the thermal effect of bulk water. Results are promising for promoting the developments of advanced nanofluidic systems and terahertz technology for physiologic research	[38]
Membrane	0.1	33 mW/cm^2^	20 min	THz irradiation facilitates the transmembrane transport of small molecules, stimulating both cellular endocytosis and diffusion processes, which led to a significant increase in Rh123 fluorescence in the cytoplasm A decrease in irradiation power density from 33 to 7.8 mW/cm^2^ demonstrated that the power density is the determining parameter for the influence of THz on the behavior of the cell membrane permeability	[39]
	3.10, 2.42	–	20 min	Fluorescence polarization analysis showed that THz radiation significantly increases cell membrane fluidity and phagocytosis of macrophages RAW264.7 without any thermal effects. The 3.10-THz group showed a more vigorous phagocytosis than the 2.42-THz group. The THz spectral characteristics of four phospholipid bilayers demonstrated a common absorption peak around 3.0–3.1 THz that significantly increased the cell membrane fluidity and phagocytosis	[40]
	2.3	230 mW/cm^2^	15 min	Analysis of the data shows that a specific feature of the response of the thermophilic bacterium *G. Icigianus* to THz irradiation was a disruption of the activity of the electron transport chain, cellular metabolism and some components of the translation system. This observation was confirmed by the rapid and sustained activation of cellular systems that compensate for the inhibition of the respiratory and glycolytic systems, namely an increase in the level of ATP synthase and a wide range of enzymes that generate NADH	[41]
Protein conformation	0.1	16 mW/cm^2^	10 min	The collective intermolecular dynamics of protein and water molecules that overlap at subterahertz frequencies is relevant to the expression of protein functions. Dielectric relaxation rate measurements were used to study the effects of externally applied electromagnetic fields on fast collective dynamics and on much slower chemical processes in protein–water systems. An aqueous solution of lysozyme, the hydration of which was not thermally balanced, was studied. It was demonstrated that subterahertz irradiation gradually reduces the dielectric constant of a lysozyme solution by reducing the orientational polarization of water molecules	[42]
	0.5 (CW) 0.1–6.0 (pulsed, 100 MHz)	6.8 mW/cm^2^ 0.4, 0.8 mW/cm^2^	0.4 s 0.4 s	Studies of protein systems with different morphology (BSA 2.5, 5.8, 10.9 mg/mL) and microtubules did not show structural changes within the sensitivity limits of the small angle X-ray scattering (SAXS) method with the used parameters of non-thermal THz irradiation with CW and pulsed (100 MHz) modes. The exposure time within 50–200 ms was chosen to collect single SAXS curves, the residence time of the current sample in the THz spot was ~ 0.4 s	[18]
	0.46	0.57 mW/cm^2^	20 min	In vitro irradiation of actin with THz waves of low intensity in a quasi-CW mode (pulse duration of 10 ms and a repetition rate of 1 Hz) leads to modulation of actin polymerization. After irradiation for 20 min, the number of actin filaments (F-actin) increases 3.5 times, which demonstrates the ability to regulate many biological functions of the cell, including its division	[27]
DNA and RNA	0.10, 0.28, and 0.46	250 mW/cm^2^	−	For samples irradiated at 0.10 THz, no significant difference was observed; radiation does not induce DNA double-strand breaks (DSBs), detected as foci of phosphorylated histone H2AX (γH2AX) used as a biomarker of cellular response to DSBs at monitoring DNA damage and repair. Instead, irradiation at 0.28 and 0.46 THz dramatically reduces the number of DSBs	[43–45]
	0.8 (pulsed)	0.5 MV/cm	60 min	Picosecond pulses were applied for 60 min at a repetition rate of 1 kHz over the entire 1 mm diameter cell culture area of human pluripotent stem cells. Using RNA sequencing of global gene-expression, it was shown that in the process of exposure of THz radiation the intracellular concentration of Zn^2+^ metal ions changes	[46]
	3.1	0.18 mW/cm^2^	2, 4, and 6 h	The long-term exposure to the low intense THz radiation significantly stimulates the process of aggregation of Aβ42 monomers, and inhibits the fibrillation phase of aggregation of Aβ42 oligomers	[47]
	0.2–3.0 (pulsed)	–	–	Cytosine (C) and cytosine monohydrate (C-MH) are representative biomolecules for studying the influence of hydrogen bonding in DNA. THz-TDS in the range 0.2–3.0 THz has been used to investigate the low-frequency vibrational modes of C and C-MH. When analyzing the nature of the modes, it was found that the overall absorption peaks of C and C-MH consist of collective vibrations mixed with intermolecular and intramolecular vibrations and that the most intense peaks of both substances are associated with prominent intermolecular translational vibrations. The THz-TDS method of C and C-MH biomolecules can be used to monitor the disruption of hydrogen bonds in DNA under high-intensity or long-term exposure to THz radiation within the theranostic approach	[48]
Neurons	0.16 0.17	10 mW 50 mW	6 min 60 min	Does not have a negative effect on the development of neurons in the hippocampus and cerebral cortex	[49–51]
	Not specified	70 μW/cm^2^	15 min, 3 h	Promotes synapse formation in cortical neurons in C57 mice	[52]
	0.094	310 mW/cm^2^	3 min	Enhancement of the growth rate of *Xenopus laevis* embryonic neurons	[53]
	0.05–1.2	0.23–11.6 μW/m^2^	3 min	Stimulation or inhibition of neuronal process growth in chick spinal cord neurons at 10–12 days	[54]
	0.1–2.0	1.1 μW/cm^2^	3–5 min	Stimulation of growth and development of neurons in the spinal cord of a chicken embryo	[55]
	0.09–0.16	Not specified	1–20 min	Neuronal atrophy due to dehydration in adult rats	[56]
	3.67	15–20 mW/cm^2^	60 min	Inhibition of neuronal growth	[57]
	0.05–2.0	0.5–50 μW/m^2^	3 min	Growth of sensory neurons in the chick embryo	[58]
	0.138	–	20 min/day, 3 days	The radiation does not cause the death of neurons and does not disrupt the nature of their internal growth; it promotes the dynamic growth of the cytosol and processes of neurons; it improves the functioning of the hippocampus and stimulates the growth of neurons	[59]
	0.1–2.0	100 μW	3 min/day, 3 days	Short-term cumulative exposure (3 min/day, 3 days) to broadband THz radiation (0.1–2 THz, with maximum energy in the region of 0.3–1 THz and maximum radiation power of 100 μW) does not cause neuronal death. It has been shown that this irradiation protocol can promote the growth of cytosomes and neuronal processes. The amount of growth in neuronal area was significantly higher in the irradiation group (22.6 ± 1.6 μm^2^) than in the control group (8.9 ± 1.5 μm^2^) on the first day. On days 2 and 3 of irradiation, similar differences were also observed, but were not statistically significant	[60]
Cancer cells	0.14	7.4 mW/cm^2^	15 min	A comparative study of the viability of normal and cancer cells when irradiated with a beam of CW THz radiation. After 15 min of irradiation, on average, only 63% of LNCaP prostate cancer cells remained alive. THz radiation can result in the death of human normal and malignant cells with different efficiency	[61]
	1.6 (pulsed, filtered with 0.57-THz FWHM)	10.4 mW/cm^2^ (maximal)	30 min	The degree of global DNA methylation in the irradiated SK-MEL-3 melanoma cells was reduced by approximately 19% compared to control (non-irradiated) cells. The degree of demethylation increased with increasing THz radiation power density. Terahertz demethylation downregulates the FOS, JUN and CXCL8 genes, which are involved in cancer cell formation and apoptosis	[45]
	0.2–3.0 (pulsed)	21 GW/cm^2^	30 min	Human skin fibroblast, neuroblastoma, and glioblastoma cell lines were used as a cell model. The genotoxic effect of THz radiation was assessed using quantitative analysis of histone H2AX phosphorylation foci. The results show that high-intensity broadband THz pulses do not cause genotoxic effects, with the genotoxicity threshold for fibroblasts ranging from 21 to 32 GW/cm^2^	[62]
	0.094	3.1, 7.8, and 18.6 kW/cm^2^	60 min	The structure of the neuronal actin protein is compromised in mouse embryonic cancer cells	[63]
	0.46	600 mW/cm^2^	30, 60 min	An increase in cytoplasmic F-actin has been demonstrated in vivo in human HeLa cancer cells under the influence of low-intensity quasi-CW THz irradiation (pulse duration of 10 ms and a repetition rate of 1 Hz), which stops cell division during cytokinesis	[64]
	4.0 (pulsed, macropulse, ~ 100 micropulses of 5 ps)	80, 160, and 250 μJ/cm^2^/micropulse	30 min	In vivo irradiation of HeLa cells with high-intensity pulsed THz waves. After irradiation at a fluence of 80 μJ/cm^2^/micropulse for 30 min (16 mW/cm^2^ of peak power), the number of actin filaments (F-actin) decreases by almost half	[28]
	83 (3.6 μm, 10 ns pulse duration, 10 Hz repetition rate)	0.3–5.0 mW	30 min	The migration of HCT116-Luc cancer cells (human colorectal carcinoma cell line expressing luciferase) is inhibited by 50% and glycolysis is reduced by 60% at THz modulation- (THM) treatment, which is a promising nondamaging EMR intervention approach. For THM in vivo, a 60% reduction in liver metastases was found in a mouse metastatic xenograft model, successfully confirming the inhibition of cancer cell migration	[4]
	0.12–0.18 (10 MHz frequency span at central 0.15 THz)	Average power density 3.2 mW/cm^2^	1–5 min	A dose-dependent cytotoxic effect of THz radiation on rat C6 glial cells was demonstrated. After one minute of exposure, a relative number of apoptotic cells increased by a factor of 1.5, after 5 min it became 2.4 times higher than the initial value. During the exposure, the temperature change did not exceed 0.1 °C	[65]
	0.3–19.5 (pulsed)	30 mW/cm^2^	1–10 min	The mechanism of increased permeability of cell membranes under the influence of THz radiation is electrokinetic in nature. As confirmed by biological assays, pheochromocytoma PC 12 cells remained viable and physiologically healthy when exposed to THz radiation for 10 min at a temperature of 25.2 ± 0.4 °C, which caused only a temporary increase in cell membrane permeability	[66]
	0.1–3.0 (pulsed, 95 ± 5 fs)	21 GW/cm^2^, 2.8 MV/cm	30 min	Despite the high energy parameters of the THz radiation within the focused beam spot of ~ 480 μm, due to the strong attenuation of the radiation in the experimental setup, only the maximum THz pulse energy of 10 μJ reached the cells under study. The increase in temperature of the cell culture in the THz spot did not exceed 2.8°C. It was found that the proliferative activity of both normal and SK-N-BE (2) human neuroblastoma cells did not change after 30 min of exposure to THz radiation	[67]

**Table 2:** Cancer cell monitoring and imaging using THz radiation

Cells	Frequency (THz)	Effects	References
Basal cell carcinoma (BCC)	0.4–1.6	This work provides the THz characterization of the 3-D organotypic model of BCC and introduces a new set of data in exploring the effect of AG1478 anticancer drug on the refractive index, absorption coefficient, and complex permittivity of the samples (with THz-TDS)	[69]
	0.05–4	The results of a spectroscopic study comparing the terahertz properties (absorption coefficient and refractive index) of excised normal human skin and BCC are shown. Both the absorption coefficient and refractive index were higher for skin that contained BCC. The difference was statistically significant over the range 0.2 to 2.0 THz (6.6 to 66.6 cm^−1^) for absorption coefficient and 0.25 to 0.90 THz (8.3 to 30 cm^−1^) for refractive index. The maximum difference for absorption was at 0.5 THz (16.7 cm^−1^). These changes are consistent with higher water content. For tumor cells, the refractive index, absorption coefficient and dielectric constant were determined. This work contributes to the understanding of the interaction of THz pulses and tissue and explains the differences observed between carcinomas and normal tissue in THz imaging of skin, which may lead to improvements in THz technology for medical applications such as tumor delineation	[70]
Cervical cancer	0.49, 0.71, 1.04, 1.07, 1.26, and 1.37	The characteristic THz absorption peaks of in vitro cultured live HeLa cervical cancer cells were obtained from the measured THz THz-TDS spectra	[71]
Mammary cancer	0.108–0.143	A method has been developed for the preliminary diagnosis of breast cancer smaller than 1 mm^3^, which is much more effective than the current detection limit of X-ray mammography 2 mm^3^	[72]
Glioma	0.2–1.4 (pulsed)	Using THz-TDS, the ability to predict the mutational process of isocitrate dehydrogenase (IDH) for the molecular classification of gliomas has been demonstrated. 92 frozen sections of glioma tissue from nine patients were included, and terahertz spectroscopy data on absorption coefficient, dielectric loss factor, extinction coefficient, dielectric loss tangent, dielectric constant and refractive index were obtained. A model for predicting IDH mutation status in gliomas was established using Least Absolute Shrinkage and Selection Operator (LASSO), Principal component analysis (PCA), and Random Forest (RF) algorithms	[73]
U87 glioblastoma	0.6–1.8 (pulsed)	Using THz-TDS, absorption spectra of mouse blood serum in frequency range 0.6–1.8 THz in the course of experimental U87 glioblastoma growth were studied. The absorption coefficient of blood serum of mice on 21st day of tumor growth was significantly lower than for water and blood serum of mouse control group. Analysis of complex dielectric permittivity indicated an increase in amount of bound water in blood serum with tumor growth	[74]

Studying the nature of the propagation of THz radiation through tissues and biological media is of fundamental importance for further research not only into the mechanisms of the effects of radiation, but also into the problems of its safety for living organisms. The different level of hydration of cells and cellular structures make it possible to propose a label-free THz method for probing bacterial colonies, for example, to determine the viability of bacteria [68].

**Fig. 2:**
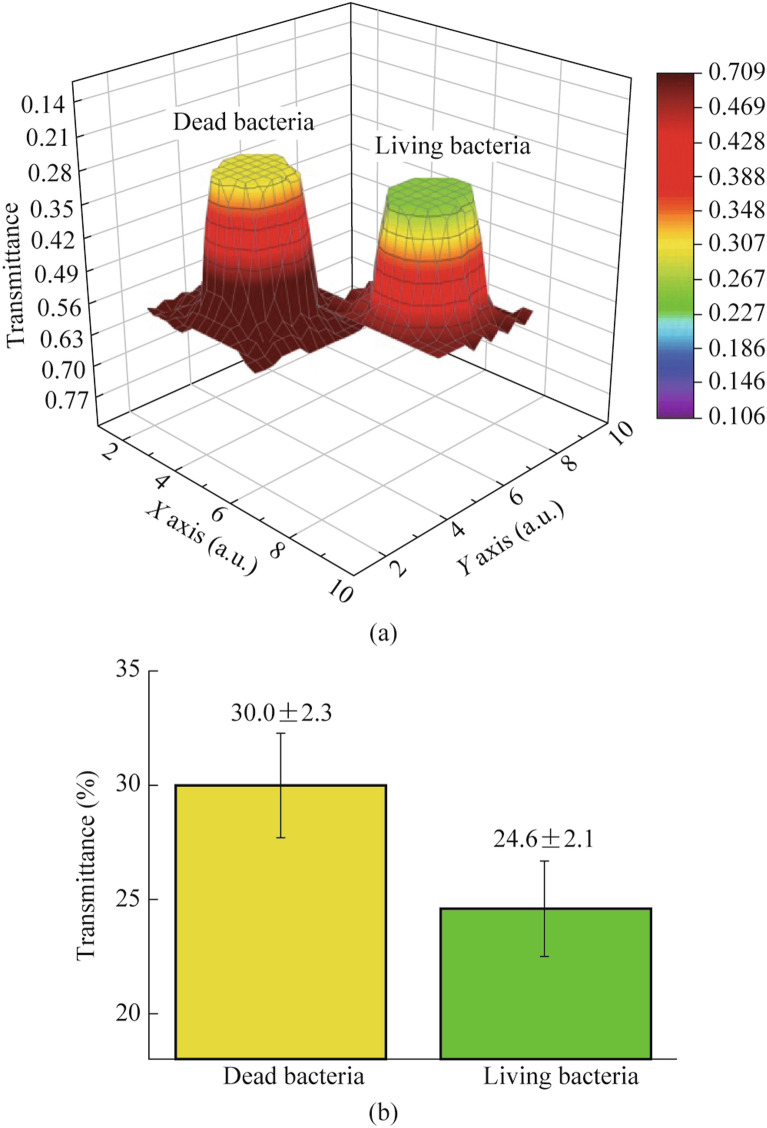
THz images of living and dead bacteria. **a** Comparison of terahertz images of live and dead *Staphylococcus aureus* cells [75]. **b** Statistical analysis of the average transmittances of the 2 groups after repeated measurements (*n* = 15). Data are presented as mean ± standard deviation. For statistical comparison of two groups, an independent *t*-test was used (*P* < 0.05)

The authors used a CW generator with an output power of 40 mW at a frequency of 2.52 THz and a detector based on a Golay cell. Denaturation of proteins during thermal treatment of bacterial cells is accompanied by changes in their THz transmission images due to modification of the vibrational–rotational response of biomolecules and their interaction with water molecules (Fig. 2) [75]. Since water molecules are closely associated with metabolic activity, changes in the bound/free water concentration ratio are responsible for significant differences in the absorption of THz radiation by living and dead bacteria *Staphylococcus aureus* and *Bacillus thuringiensis*. In this context, it is interesting to note that in [76] it was proposed to use the microwave dielectric response of water as a marker of changes in glucose levels in erythrocytes.

**Fig. 3:**
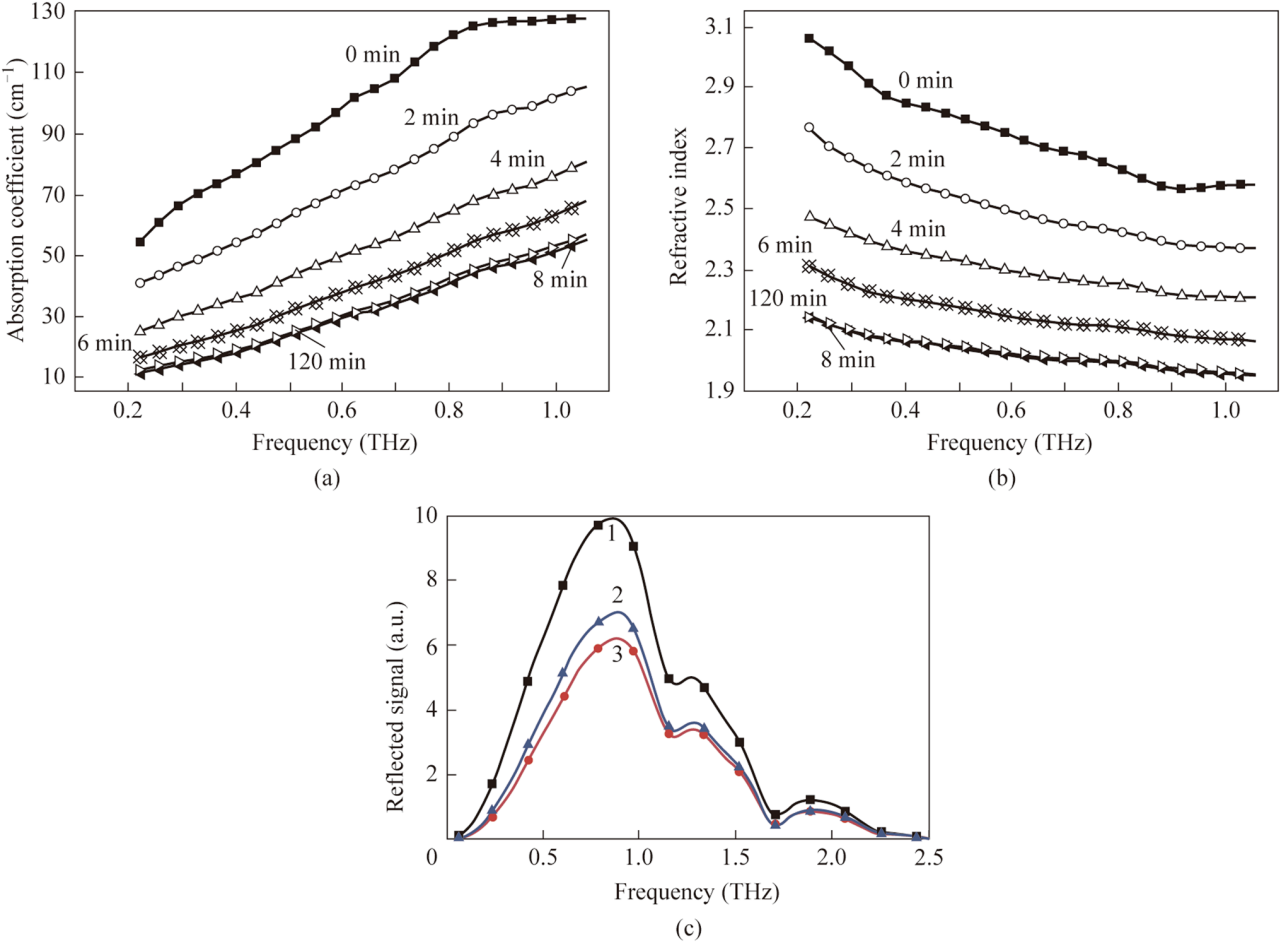
Spectral dependences of the **a** absorption coefficient and **b** refractive index of the bovine muscle tissue treated with 99.9% glycerol. The time corresponds to the cumulative time of holding the sample with glycerol. **c** Spectrum of the reflected signal from a total internal reflection prism in air (1) and for bovine muscle tissue (2, 3) under the influence of propylene glycol for 3 min (3) and 90 min (2). The signal from the tissue gradually increases with time, indicating a loss of water in the prism-sample interface and an increase in the propylene glycol content in the tissue [77]

On the other hand, strong absorption by water molecules prevents THz waves from penetrating highly hydrated tissues and probing biological molecules in aqueous solutions. In [31], the authors discuss approaches to overcome these shortcomings—new methods of freezing and temporary dehydration of tissues using their drying or the action of hyperosmotic agents. The use of such techniques has the potential to probe tissue to sufficient depth to identify and characterize superficial cancers. As a result, reflected THz radiation can provide the necessary contrast between healthy and pathological tissues for reliable marking of the tumor border, which is certainly important from the point of view of the use of THz waves in medical practice. The work [77] also analyzed the main reasons for the small probing depth and low contrast of THz images of pathological tissue inhomogeneities and made experimentally confirmed proposals for increasing the probing depth and image contrast using hyperosmotic immersion optical clearing agents (OCAs). The authors concluded that the most preferred OCAs are highly concentrated (99.9%) glycerol and propylene glycol (Fig. 3). For fresh muscle tissue, the absorption coefficient at a frequency of 1 THz decreases from 130 to 50 cm^–1^, and the refractive index from 2.60 to 1.95, after 8 min of exposure to glycerol.

It can be concluded that the solution to the problem of delivering THz radiation lies in the temporary and reversible dehydration of living biological tissue, which leads to a multiple (up to 10 or more fold) reduction in the amount of absorbed radiation energy in the cleared tissue and a significant decrease in the Fresnel reflection from its surface due to 1.5–2-fold reduction in refractive index.

In [19], an existing shortcoming in physical models for describing the absorption of THz radiation by tissues under normal conditions and pathology was identified; it was shown that corrected models should be used to describe the dielectric constant of brain tissue. In particular, it was initially difficult to determine the exact boundaries of edema in cases of traumatic brain injury and the presence of a tumor. To address this shortcoming, it is proposed to use two models to analyze WHO grades I–IV gliomas and healthy brain tissue, using complex dielectric constant to describe the experimental response of both tissues measured ex vivo. Physico-mathematical models of the effective terahertz complex dielectric constant of brain tissue have been developed [78–83]. They are important for the analysis of elementary electric dipole excitations that underlie the effective dielectric response of tissues, as well as for subsequent modeling of the interaction of radiation with tissues in the development of diagnostic tools and methods. This problem is solved using two models.

• The first is a model of two Debye relaxers (double Debye model—DD):

1 $$\widetilde{\varepsilon }={\varepsilon }_{\infty }+\frac{{\Delta }_{\varepsilon 1}}{1+\text{i}\omega {\tau }_{1}}+\frac{{\Delta }_{\varepsilon 2}}{1+\text{i}\omega {\tau }_{2}},$$

widely used in terahertz biophotonics, where *ε*_∞_ is the high-frequency dielectric constant (at significantly higher frequencies compared to the spectral position of the Debye absorption bands), *τ*_1_, *τ*_2_ and Δ*ε*_1_, Δ*ε*_2_ are the times and amplitudes of “slow” and “fast” relaxations. This model describes two broad absorption bands on the curve *ε*′′, related to the “slow” (*τ*_1_) and “fast” (*τ*_2_) relaxations of water with maxima at frequencies *ν* = (2π*τ*_1_)^−1^ and (2π*τ*_2_)^−1^. The function *ε*′ decreases with increasing frequency and has the values *ε*′(*ν* → 0) = *ε*_∞_ + Δ*ε*_1_ + Δ*ε*_2_ and *ε*′(*ν* → ∞) = *ε*_∞_ in the low- and high-frequency limits. The DD model provides a convenient parameterization of the effective optical characteristics of tissues using 5 coefficients – *ε*_∞_, Δ*ε*_1_, Δ*ε*_2_, *τ*_1_, and *τ*_2_.

• The second is a model of two overdamped-oscillator Lorentz oscillators (double overdamped-oscillator model – DO) –

2 $$\widetilde{\varepsilon }\text{=}{\varepsilon }_{\infty }+\frac{{\Delta \varepsilon }_{1}}{1-\frac{{\omega }^{2}}{{\omega }_{01}^{2}}+\text{i}\frac{\omega {\gamma }_{1}}{{\omega }_{01}^{2}}}+\frac{{\Delta \varepsilon }_{2}}{1-\frac{{\omega }^{2}}{{\omega }_{02}^{2}}+\text{i}\frac{{\omega \gamma }^{2}}{{\omega }_{02}^{2}}},$$

was first used to describe the terahertz response of biological tissues, where Δ*ε*_1_, Δ*ε*_2_ are the amplitudes of two Lorentz oscillators, *ω*_01_, *ω*_02_ are quasi-resonant frequencies, *γ*_1_, *γ*_2_ are attenuation parameters. Two oscillators with amplitudes Δ*ε*_1_ and Δ*ε*_2_ are almost identical to the “slow” and “fast” Debye relaxations in Eq. (1). The constant *ε*_∞_ and the amplitudes of the Debye relaxers Δ*ε*_*i*_ in the DD model are equivalent to those in the DO model. Other parameters of the *i*-th overdamped oscillator can be uniquely calculated based on the parameters of the corresponding Debye relaxer by solving the system of equations:

3 $${\gamma }_{i}={\omega }_{0i}^{2}{\tau }_{i}, {\gamma }_{i}=C{\omega }_{0i}, C\gg 1.$$

When the inequality *C* ≫ 1 is satisfied, the specific value of the constant *C* is not important; it was *C* = 10^2^. In an overdamped oscillator, the parameters *ω*_0_, *γ*_*i*_ lose their independent physical meaning, and their combination *ω*_0_, *i*/*γ*_*i*_≈*τ*_*i*_^−1^ determines the position of a wide absorption band in the spectrum *ε*′′.

The Debye relaxer and the overdamped oscillator are equivalent in the low frequency region (down to *ω*∼(2π*τ*_*i*_)^−1^ < *ω*_0,*i*_ ≪ *γ*_*i*_), but in the high frequency region (*ω* > (2π*τ*_*i*_)^−1^) the Debye model predicts higher losses *ε*′. Unlike an overdamped oscillator, the Debye kernel does not satisfy the sum rule for oscillator strengths. In turn, integrating the conductivity given by the *i*-th overdamped oscillator gives the final number

4 $$\Delta \varepsilon_{i} \omega_{0}^{2} = \Delta \varepsilon_{i} \gamma_{i}^{2} /C^{{2}} ,$$

where *ω*_0,*i*_^2^ = *N*_*i*_*q*_*i*_^2^/(*m*_*i*_*ε*_0_), *N*_*i*_ is the number of charges/dipoles participating in the *i*-th relaxation process, and *q*_*i*_ and *m*_*i*_ are the effective charge and mass. To correctly describe the spectra of *ε*′ and *ε*′′ in the high-frequency region (*ω* > (2π*τ*_*i*_)^−1^) and quantify the number of charges/dipoles in the medium *N*, it is preferable to use a more rigorous DO model. This makes the DO model more suitable for terahertz biophotonics. DD and DO models are applied to analyze the results of ex vivo terahertz pulsed spectroscopy of brain and other tissues. The image contrast in the THz range is caused by the non-uniformity of the absorption coefficient and refractive index values, which differ due to the different content of water molecules in malignant tumors and normal tissue.

The penetration depth of THz radiation turned out to be generally sufficient for the study of carcinomas [31, 77], which most often arise on epithelial tissues, but in general the problem of ensuring the necessary depth of radiation penetration for diagnostic purposes and exposure to deep-lying cellular structures remains [84, 85]. One of the effective solutions to this problem is temporary and reversible tissue dehydration using OCAs [77, 79, 86].

Note that modern research in the field of tumor imaging technologies in the THz range uses data analysis algorithms based on machine learning, including deep learning, which allows increasing the accuracy and reliability in differentiating healthy and tumor tissue [87–89].

From the point of view of the safety of THz radiation, the International Commission on Non-Ionizing Radiation Protection (ICNIRP) limited the safe intensity of exposure of the population to no more than 1 mW/cm^2^ in the frequency range from 2 to 300 GHz for 6 min [90]. This safety limit is based on proven thermal effects associated with the main effects in this frequency range. However, the biological effects of THz radiation are recorded at radiation power flux densities significantly lower than 1 mW/cm^2^. At such a low radiation intensity, the integral heating of irradiated objects in the experiment does not exceed 0.1 °C [91]. Above the frequency of 300 GHz there are no established limits for public exposure. Permissible levels of radiation from transmitting radio equipment in sanitary and residential areas (uncontrolled exposure, for the population) vary markedly in some countries (higher levels at higher frequencies) [92] (Table 3).

**Table 3:** Permissible levels of radiation from transmitting radio equipment in the sanitary and residential zones of a number of countries [93]

Country	Permissible intensity level (µW/cm^2^)
Russia, Poland, Belarus, Kazakhstan	10
Ukraine	100
USA, Europe (except some countries), Japan, Republic of Korea	200–1000
Canada	130–2000
China	10 (40)–2000

These maximum intensity levels are determined based on the thermal effects that occur when exposed to EMR on living objects. However, analysis of modern data on the mechanisms of action of THz radiation provides grounds for the need to introduce new safety standards for the use of THz radiation, which is due to the identification of selective effects when it is absorbed by individual biological targets in a living organism, for example, DNA in the form of gene expression and genetic consequences of exposure. For example, in [14–16], with pulsed irradiation of living cells by a free electron laser (NovoFEL) of the Institute of Nuclear Physics of the Siberian Branch of the Russian Academy of Sciences (Novosibirsk, Russia) at a frequency of 0.14 THz with the average power density ~ 2 mW/cm^2^ and exposure of 15 or 30 min, changes in the activity of the promoter of several genes were recorded.

It is quite obvious that the issues of propagation and penetration of THz radiation in biological media and tissues are important not only for the diagnosis and mapping of tumors, but also for studying the consequences of exposure to radiation and determining the correct safety boundaries.

### Impact of THz radiation on cell membrane permeability

Many physiologic processes in living organisms are closely related to the permeability of ion channels on the cell membrane, such as maintaining cell membrane potential, generating action potentials, conducting nerve signals, regulating the central nervous system, heartbeat, skeletal muscle contraction, hormone secretion, and so on. Abnormal permeability of ion channels usually leads to various diseases, such as cardiovascular and cerebrovascular, neurodegenerative, and tumor [94, 95].

**Fig. 4:**
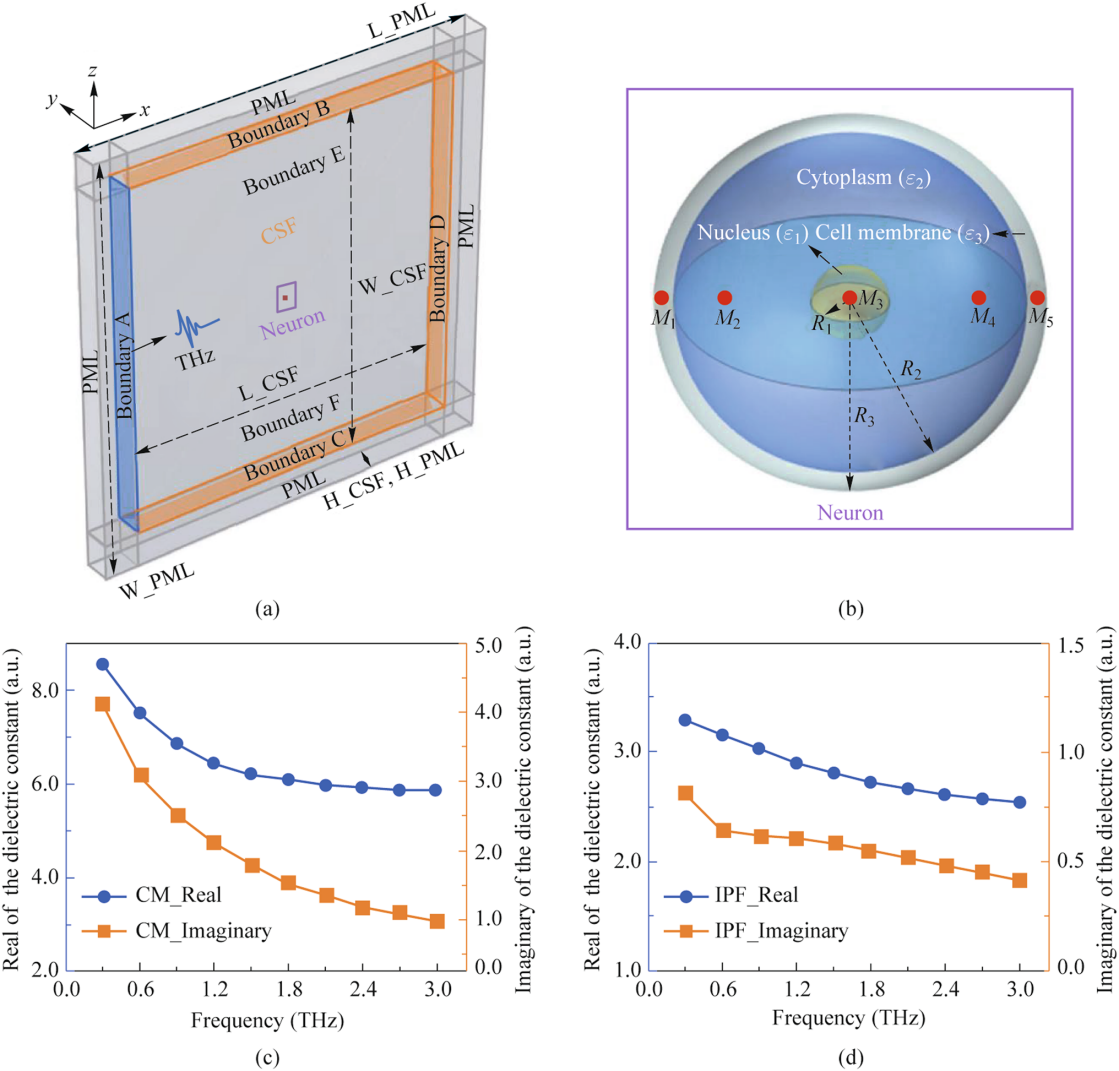
Simulation model and dielectric constant of nervous tissue. **a** Terahertz wave transport and thermal effect model in the nervous tissue, including neurons, terahertz sources, cerebrospinal fluid and PML layers. **b** A three-dimensional neuronal model consisting of a nucleus, cell membrane (CM) and cytoplasm. The red area is the location of the sampling point (M1–M4). **c** The real and imaginary parts of the relative permittivity of the CM (3–3 THz). **d** The real and imaginary parts of the relative permittivity of the intracellular physiologic fluid (IPF). A perfectly matched layer (PML) is an artificial absorbing layer for wave equations, commonly used to truncate computational regions in numerical methods to simulate problems with open boundaries, especially in the FDTD and FE methods. The key property of a PML that distinguishes it from an ordinary absorbing material is that it is designed so that waves incident upon [60]

The neuron membrane consists of a phospholipid bilayer, which forms the main cytoskeleton [96]. The work [60] shows how the frequency and power of terahertz waves affect the electrical field strength and temperature in nerve cells. It has been established that there is a positive correlation between them; in other words, a decrease in radiation power reduces the increase in temperature in neurons (Fig. 4).

Figure 4 shows how terahertz waves first enter the cerebrospinal fluid, the pathway is approximately 10 μm through the cerebrospinal fluid, then they enter the neuron. Ultimately, the terahertz waves are completely absorbed by the perfect matching layer (PML).

To simplify, a circular neuron model was created with a three-layer structure containing cell membrane, cytoplasm, and nucleus (Fig. 4b). The red areas in the figure indicate the locations of the sampling point, which are used to analyze the distribution of terahertz waves in different regions of neurons. It turned out that short-term cumulative exposure (3 min/day, 3 days) to broadband THz radiation (0.1–2 THz, with maximum energy in the region of 0.3–1 THz and maximum radiation power of 100 μW) does not cause neuronal death.

**Fig. 5:**
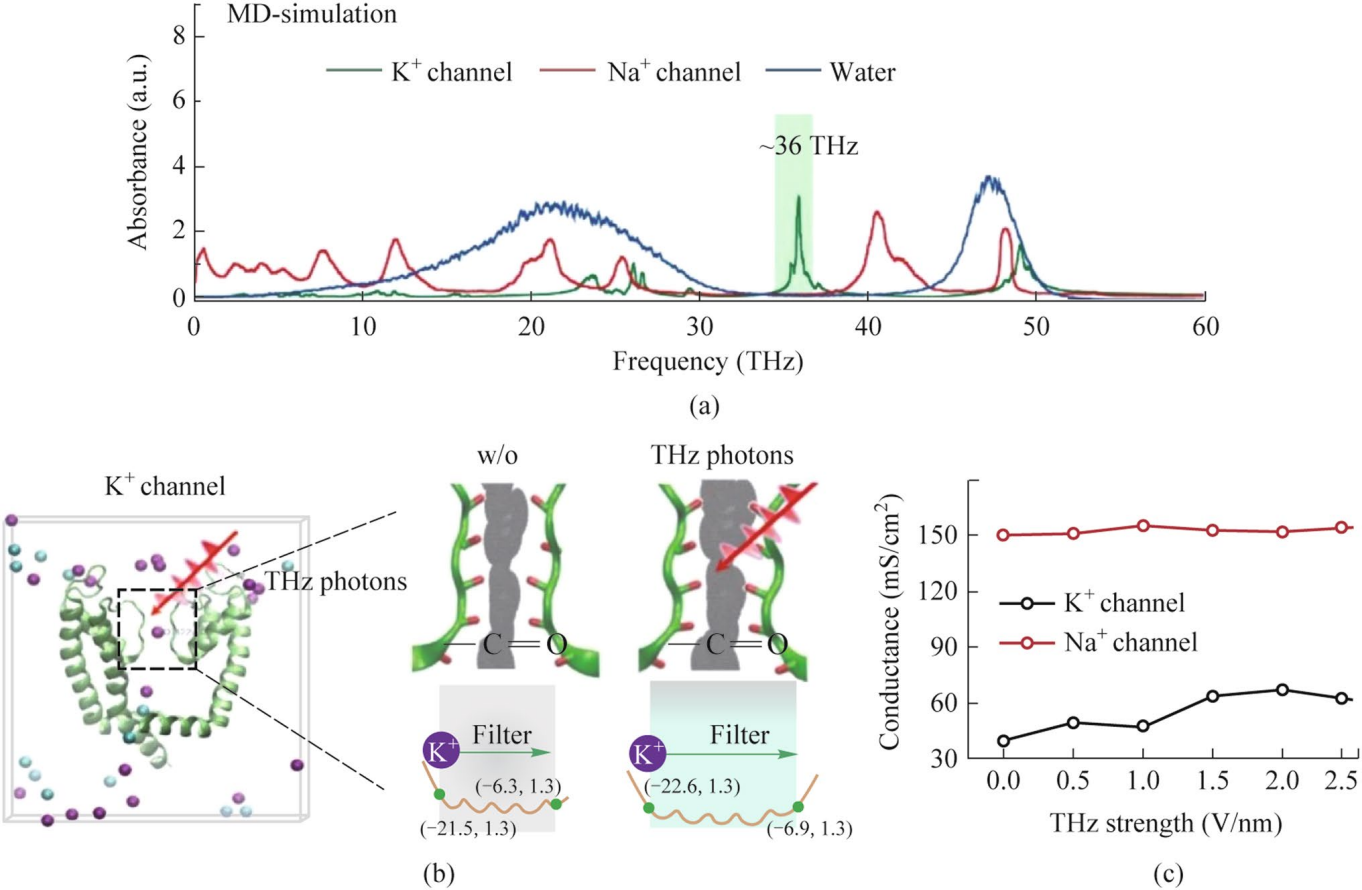
Specific frequency THz photons resonate voltage-gated potassium (Kv) channels and decrease the action potential (AP) firing rate in cortical neurons through molecular dynamics simulation. **a** Absorbance spectra of voltage-gated potassium/sodium ion channels and the bulk water. **b** The dynamic attributes of the low-threshold Kv1.2 filter structure in pre- and post-exposure to HFTS. Purple balls represent the K^+^, blue balls represent the Cl^−^. **c** The alterations in potassium/sodium ion conductance consequent to the influence of HFTS [95]

The resonant nature of the absorption of THz by a nerve cell and confirmation of the existence of non-thermal effects was carried out in [95]. In particular, it was shown that high-frequency terahertz stimulation (HFTS) in the vicinity of a frequency of 36 THz can suppress the activity of pyramidal neurons by increasing the conductance of voltage-gated potassium channels (Fig. 5a).

The results obtained showed a significant change in the permeability of the –C=O molecular groups in the channel filter structure, as evidenced by an increase in the van der Waals radius of the channel by approximately 0.5 Å (Fig. 5b). During THz exposure, the potassium ion channel conductance increased almost linearly with THz field intensity, while the sodium ion conductance remained largely unchanged (Fig. 5c). The results obtained indicate that terahertz photons primarily weaken the excitatory activity of neurons by increasing the conductance of potassium ions, thereby modulating the excitability of neurons.

**Fig. 6:**
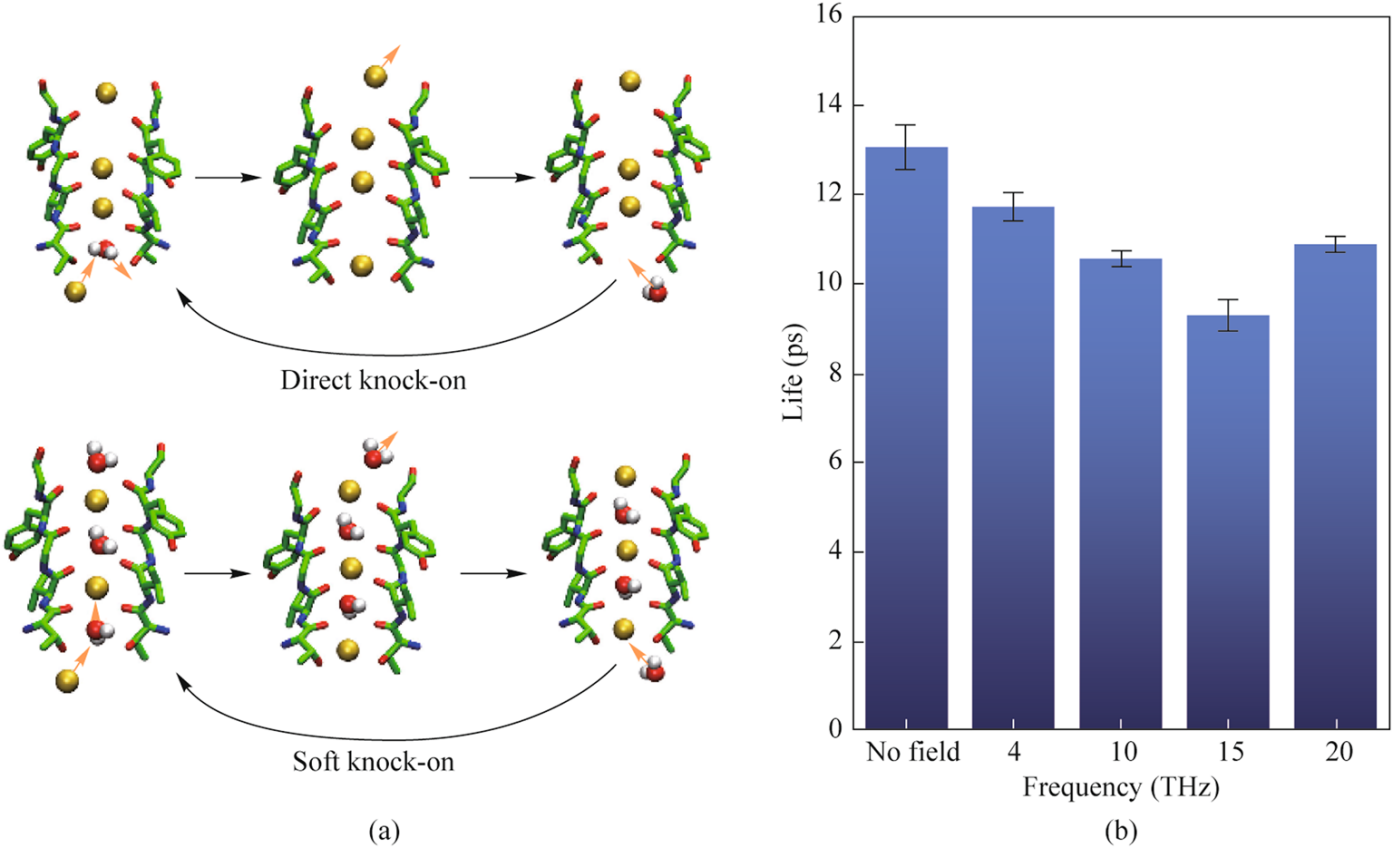
** a** Difference between the two modes of potassium ion transport. **b** Lifetime (Life) of the hydrogen bond formed by water molecules and hydroxyl oxygen atoms on the side chain 374 Threonine [97]

The work [97] presents the results of studies of the influence of external terahertz electromagnetic fields with various frequencies of 4, 10, 15, and 20 THz and an amplitude of the electrical component of 0.4 V/nm on the permeability of the potassium ion channel on the membrane of the nerve cell (Fig. 6).

It has been established that the lifetime of the hydrogen bond formed between water molecules and hydroxyl oxygen atoms in the side chain of 374 Threonine becomes the main factor controlling the penetration of ions. Especially when the frequency of the applied electric field is 15 THz, the hydrogen bond lifetime is reduced by 29%, and the ion flux in the membrane channel is increased by 67.7%. The work [97] examines several mechanisms of ion penetration and their combination: the first (“soft knock-on”) assumes that water molecules have a synergistic effect, reducing the dielectric constant and increasing the rate of ion transfer. The second (“direct knock-on”) suggests that the key to efficient conduction of K^+^ ions lies in the Coulombic interaction between neighboring ions, rather than in water molecules co-penetrating the K^+^ ions.

**Fig. 7:**
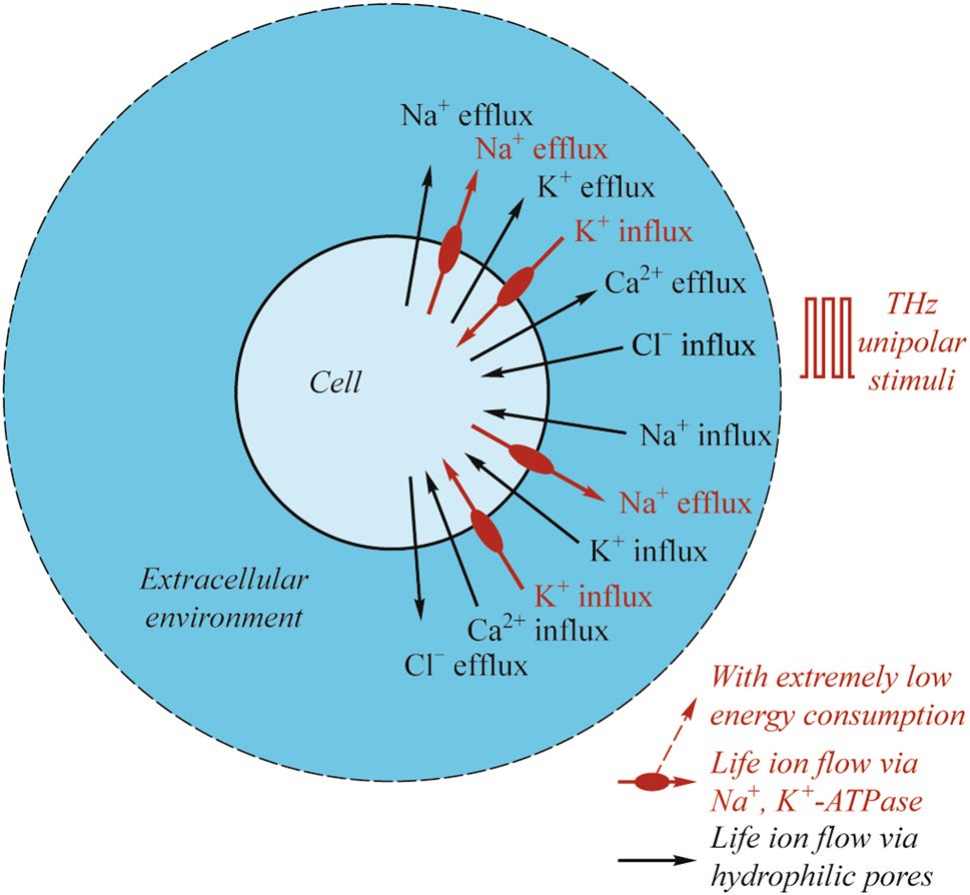
Illustration of cell system under the stimulation of terahertz unipolar picosecond pulse train [92]

The Na^+^, K^+^-ATPase (NKA), as a ubiquitous enzyme responsible for creation and maintaining Na^+^ and K^+^ gradients across the cell membrane by transporting 3 Na^+^ out and 2 K^+^ into the cell, is activated during the life of ion flows through the hydrophilic pores of the cell membrane when stimulated by a train of terahertz unipolar picosecond pulses [92]. The terahertz repetition frequency of the unipolar picosecond pulse train is respectively 0.1, 0.21, 0.3, 0.5, 0.51, 0.7, 0.9, and 1.2 THz. The duty cycle of the pulse train is 0.5 so the pulse width of each unipolar pulse in the train is 5, 2.38, 1.67, 1, 0.98, 0.71, 0.55, and 0.41 ps, respectively. After the stimulation, the NKA and hydrophilic pores can stay open for tens of nanoseconds before the close of NKA (Fig. 7).

**Fig. 8:**
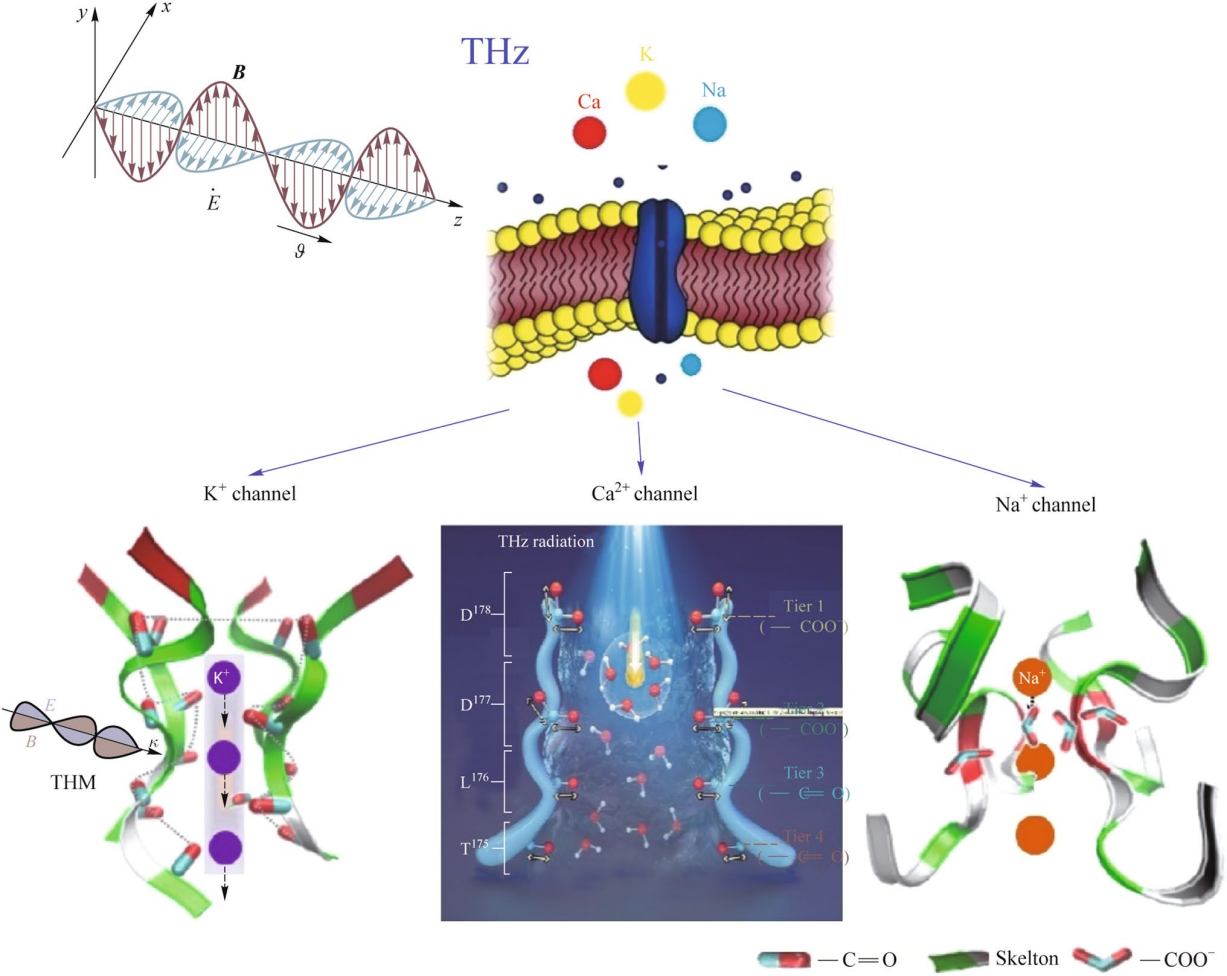
Review of the penetration of K^+^, Na^+^, and Ca^2+^ ions through the cell membrane when exposed to THz radiation, THM is the terahertz modulation, adapted from [51, 98, 99]

The life ion flow via NKA is several orders of magnitude smaller than the flow via hydrophilic pores and has opposite transmembrane transport direction to the flow via the pores at a relatively long time after the stimulation. The life ion flow via the pores and NKA causes a negligible change in intracellular Na^+^ and K^+^ concentration even at a relatively long time 80 ns after the stimulation when the NKA closes. In two different types of cells, rat neostriatal neuron and guinea-pig ventricular myocyte, the power dissipations of NKA during the life ion flow via hydrophilic pores are both at as low as around the level of 10^−11^ W. And the power dissipation becomes zero after the close of NKA. This level of power dissipation is still qualitatively far larger than the power dissipation (the level of 10^−18^ W) of cell membrane Ca^2+^ ATPase during the flow via voltage-gated calcium channels under terahertz bipolar picosecond pulse train stimulation where no hydrophilic pore is formed. This might indicate that the activation of hydrophilic pores may cause a drastic increase in energy consumption of cell metabolic energy during the information communication of cells. The results also show that the conclusions are tenable under different stimulation frequencies in 0.1–1.2 THz. The effect of THz radiation on the permeability of the cell membrane for individual ions is summarized in Fig. 8 [51, 98, 99].

It is interesting to note that the effect of THz on ion transport ultimately changes the metabolism of the entire cell and tissue. Thus, in [42], the collective intermolecular dynamics of protein and water molecules was studied in the subterahertz frequency range (0.1 THz pulses with an average power density of 16 mW/cm^2^, during 10 min of irradiation). In particular, dielectric relaxation (DR) measurements were performed to study how THz fields disrupt collective dynamics and influence slow chemical processes in protein-water systems. Thus, it was found that THz irradiation gradually reduces the dielectric constant of a lysozyme solution due to a decrease in the orientational polarization of water molecules, as a result of a slow shift toward the hydrophobic-hydrate structure of lysozyme.

### Modulation of intracellular processes induced by THz radiation

**Fig. 9:**
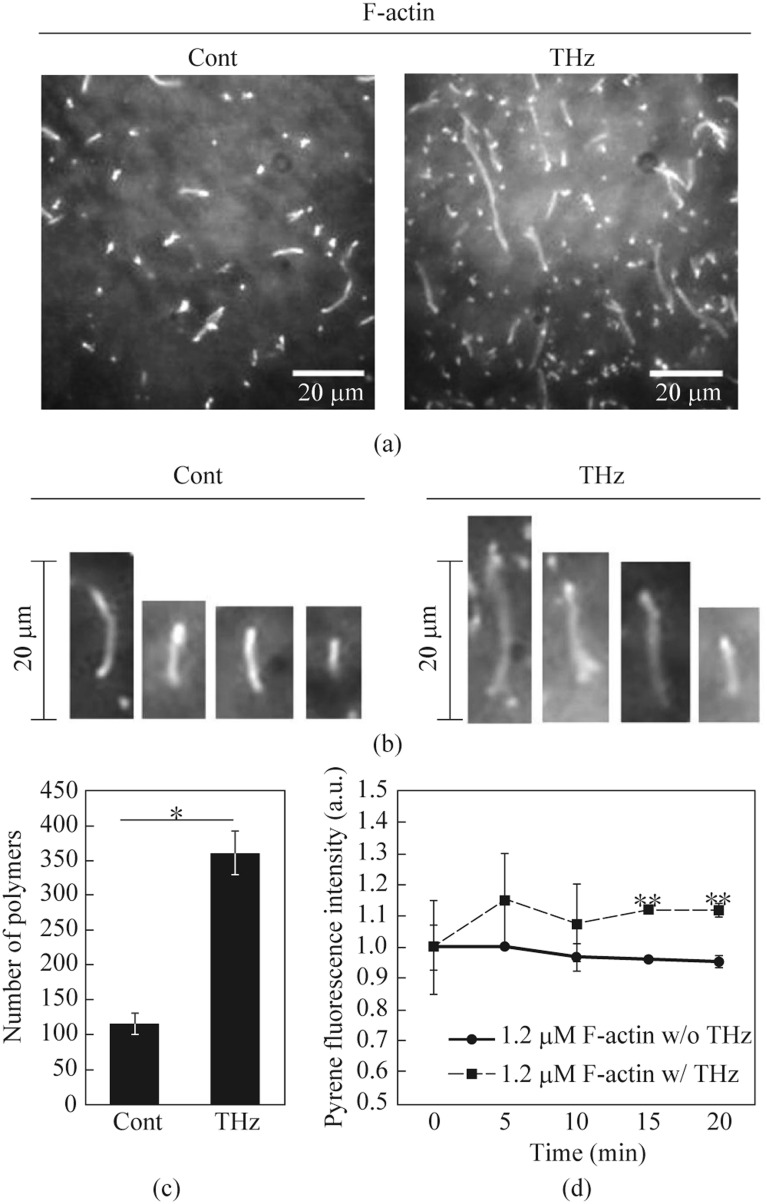
Effect of THz radiation on actin filaments [23]. G-actin solution (0.8 μM) was polymerized by adding F-actin buffer with 20 min irradiation with THz radiation (0.46 THz) and without irradiation (control): **a** Images of actin filaments; **b** Comparison of actin filament morphology; **c** Comparison of the number of actin filaments. More than 100 actin filaments were counted in each experiment, *p < 0.05; **d** Pyrene-actin filaments were formed for 1 h, and then fluorescence was measured each time with or without THz irradiation. The relative fluorescence of pyrene at 0 min was determined to be 1.0. Data shown is the average of three independent measurements, **p < 0.01 [27]

**Fig. 10:**
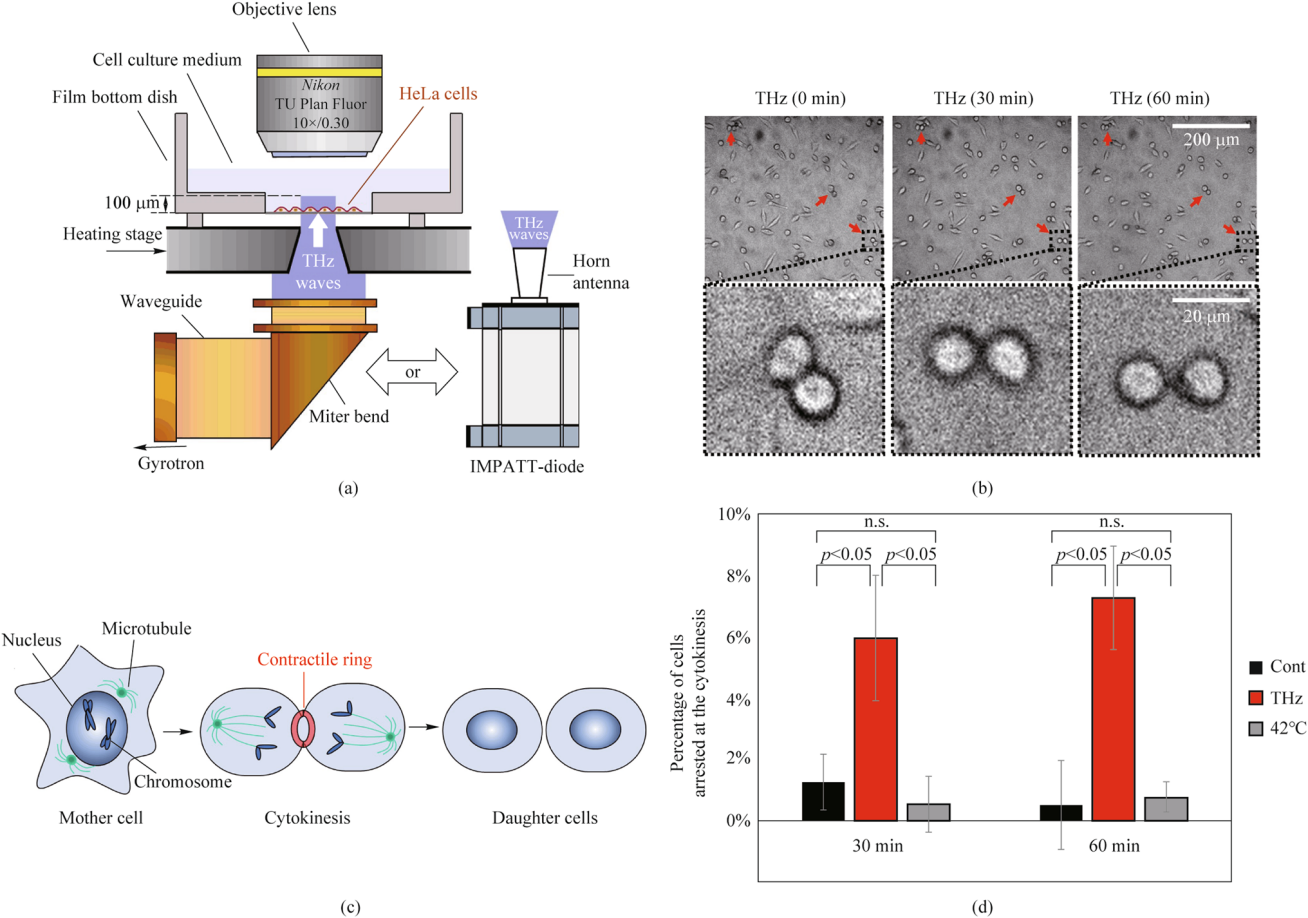
Effects of THz irradiation on HeLa cell morphology [64]: **a** Schematics of the experimental setup (THz waves were generated by a gyrotron FIR-UF on frequency of 0.46 THz with pulse duration of 10 ms and a repetition rate of 1 Hz. The THz irradiating beam passed vertically from the bottom of the dish via an aperture of 4 mm in the heating stage has a power density of 600 mW/cm^2^. Also, CW IMPATT-diode (TeraSense Group Inc) with a frequency of 0.28 THz providing a power density of 125 mW/cm^2^ on the output of horn antenna was used). **b** Microscopy images of cells at 0, 30, and 60 min of irradiation on frequency of 0.46 THz. **c** Schematics of mitotic progression. **d** Percentage of cells arrested at cytokinesis. More than 184 cells were measured in each experiment

Speaking about the evolutionary mechanisms of protecting a biological cell from EMR, it is necessary to note a number of works on the influence of THz radiation on the processes of cell division. Thus, in [27, 28] it was established that THz radiation affects the key mechanism of cell division—the formation of actin protein filaments, which is a component of the cellular framework of eukaryotic cells. The authors present the results of a series of measurements and show that EMR in the terahertz range at low energy density can accelerate the actin polymerization reaction in the cell, but do not disrupt the integrity and viability of the cell (Fig. 9). The effect of radiation from a gyrotron operating at a frequency of 0.46 THz in a quasi-continuous mode with pulses with an intensity of 0.57 mW/cm^2^, a duration of 10 ms, and a repetition rate of 1 Hz, or a fluence of 5.7 mJ/cm^2^, was studied. Polymerization of monomeric actin into filaments plays a key role in cell motility, growth and differentiation, and gene expression. The data presented in Fig. 10 demonstrate that irradiation with THz waves at a frequency of 0.46 THz leads to modulation of actin polymerization, which is monitored by recording the fluorescence of pyrene-actin fluorophores. After THz irradiation for 20 min, the number of actin filaments increases by 3.5 times, which demonstrates the ability to effectively control actin polymerization and is important for understanding and regulating many biological functions, including cell division.

It is important to note that the nature of the effect of radiation on actin changes significantly if macropulses are used at a frequency of 4 THz with a repetition rate of 5 Hz, consisting of ~ 100 micropulses with a duration of 5 ps and an intensity of 16 MW/cm^2^ each (fluence 80 μJ/cm^2^/micropulse), for which, instead of stimulating the growth of actin filaments, their destruction is observed both in vitro and in vivo for HeLa human cancer cells. In [28], it was shown that the energy of THz pulsed radiation can propagate in an aquatic environment over a distance of more than 1000 μm. The authors interpreted this effect as the propagation of shock waves induced by THz pulses, and visualized such shock waves in distilled water samples. Irradiation of a biological cell with quasi-continuous or pulsed THz radiation, the choice of frequency and energy density turned out to be fundamental factors for obtaining multidirectional biological effects that require additional research.

While it was previously shown that low-intensity THz irradiation enhances the polymerization of purified actin in vitro [64] (Fig. 9), subsequent work by the same authors demonstrated an increase in cytoplasmic F-actin in vivo in HeLa human cancer cells (Fig. 10). The paper presents a morphological analysis of HeLa cells, which showed that THz irradiation stops cell division during cytokinesis (Fig. 10b). Normally (control), at the final stage of cytokinesis, the contractile ring consisting of F-actin (Fig. 10c) should disappear in less than 15 min, but with THz irradiation it persisted for 60 min. The preservation of the paired round of cells indicates that THz irradiation inhibited the progression of cytokinesis. Thus, it is one of the key biomechanisms affected by THz waves.

One of the most important is the issue of the impact of THz radiation on DNA and the subsequent implementation of the genetic program for the development and functioning of living organisms. In [46], a setup was developed and a study was carried out on the effect of THz pulses on gene expression in living human induced pluripotent stem cells. Picosecond EMR pulses with a maximum electric field strength of 0.5 MV/cm (maximum THz signal amplitude at a frequency of 0.8 THz) were applied for 1 h with a repetition rate of 1 kHz to the entire cell culture area of 1 mm in diameter. Using RNA sequencing of global gene-expression, it was shown that many THz-regulated genes are controlled by zinc–finger transcription factors; in particular, when exposed to THz radiation, the intracellular concentration of metal ions, such as Zn^2+^, changes.

In [100], a label-free method of genetic probing using THz waves is discussed; in other words, from the spectrum of THz signals, it is possible to determine the binding state of oligo- and polynucleotides, which allows determination of the genetic composition of target polynucleotides by determining their binding to known probe molecules. The spectral features of the absorption of DNA molecules are the result of the interaction of EMR with DNA and RNA macromolecules [101]. Transmittance measurements at frequencies of 0.2–0.75 THz are reported for single- and double-stranded RNA molecules with known base pair sequences, and a method for mathematically predicting low-frequency THz absorption for short artificial DNA and RNA is presented.

It was shown in [43] that THz irradiation with a frequency of 0.1 THz (180 mW) and 0.28 THz (20 mW), a power density of 250 mW/cm^2^ and an exposure time of 10 min enhances the processes of DNA break repair due to the non-thermal effect. The work demonstrates the possibility of non-invasive therapy with THz waves of double-stranded DNA breaks.

The possibility of DNA damage by narrowband THz radiation was studied in [102, 103]. A Novosibirsk free electron laser (NovoFEL) was used to generate the THz radiation with a wavelength of 130 μm (2.3 THz). The average radiation power density was 0.14 W/cm^2^, irradiation was performed for 1 h. It is noted that irradiation did not cause structural chromosomal aberrations in human embryonic stem cells, and there was no effect on their mitotic index or morphology. In [104], the existence of a relationship between the dynamic stability of double-stranded DNA molecules and the parameters of THz radiation was mathematically shown. The greatest changes in the conformation of DNA molecules occur only through a nonlinear mechanism, requiring a spatial perturbation above a certain amplitude threshold, which is determined by the intensity and frequency of THz radiation (in the vicinity of 2.5 THz, power up to 15 mW/cm^2^, exposure time 40 ps). It is noted that the nature of the genotoxic effects of THz is more probabilistic than deterministic.

It has already been noted that the energy of THz radiation photons is insufficient to change chemical reactions, however, the presence of nonlinear resonance can lead to local changes in gene transcription. Low-intensity (average power density ~ 1 mW/cm^2^) long-term exposure (2 and 6 h) to broad-spectrum THz pulsed radiation centered at ~ 10 THz with a pulse repetition rate of 1 kHz leads to specific (rather than global) changes in the functionality of cellular DNA. Specifically, certain genes in irradiated mouse stem cell (MSC) cultures are upregulated while other genes are downregulated. Many of the MSC genes do not respond at all to the selected irradiation conditions, showing that the effect is specific. Through the frequency mixing of ultrafast fundamental and second-harmonic laser fields, a directional plasma electron current in pressurized atomic gases can be generated. In case of ultrafast lasers (100 fs) this technique is capable of producing electro-magnetic radiation at THz frequencies. Thus, a new source of high energy is obtained (1 MJ, pulse duration 35 fs, i.e., high peak power per pulse, 30 MW), an average power density of 1 MW/cm^2^, broadband THz radiation (10 THz) with a high repetition rate (1 kHz).

Studies of the dynamics of the conformation of DNA molecules precisely indicate the importance of nonlinear excitation mechanisms, which can cause local softening of polymer bonds [105]. When the excitation amplitude exceeds a certain threshold value, the effective bending stiffness becomes negative, i.e., nonlinear excitation occurs, which, even in the absence of thermal fluctuations, causes instability in the bending of the DNA chain. Moreover, with a further increase in the amplitude of nonlinear excitation, the bending instability is replaced by the collapse instability, which leads to the coiling of the circuit into a compact coil.

### Changes in protein conformation induced by THz radiation

During the last decades discussions were taking place on the existence of global, non-thermal structural changes in biological macromolecules induced by THz radiation. In [106], it was concluded that the changes in electron density in the protein are associated only with the absorption of THz radiation when the protein is irradiated at a frequency of 0.4 THz with a total power of 12 mW and an exposure time of 25 ms. The observed changes in electron density can only be explained by the collective excitation of dipole oscillators in the protein, as suggested by Fröhlich. The presence of collective vibrational excitation in biomolecules has broad implications for self-organization inside cells and for nonequilibrium thermodynamics of reactions involving protein molecules. For example, different THz radiation intensities can change the Boltzmann factor between vibrational states, making the rate of protein reactions dependent on the excitation of THz radiation rather than on the thermal equilibrium temperature.

In [82], bovine serum albumin (BSA) was exposed to continuous waves at a frequency of 3.67 THz with a power density of 20 mW/cm^2^ for 60 min. As a result, changes in the UV and circular dichroism (CD) spectra of the irradiated protein were observed. These were attributed to modifications in the BSA conformation, which was further confirmed by an increase in tryptophan (Trp) fluorescence of BSA and a twofold decrease in the progesterone binding constant. Data on the effect of 60 min exposure of pulsed THz radiation with a frequency band from 0.2 to 1.5 THz and a peak intensity of 10 mW/cm^2^ are also presented, while the sample was prepared in the form of thin films on a crystalline SiO_2_ substrate. Then the BSA was dissolved in water, where it reacted with certain adsorbates of great biological significance, that is, with oxygen, ozone and nitric oxide. THz waves have been found to cause obvious changes in such interactions. The interaction between BSA and oxygen was studied using in situ spin probing techniques. The spin probe was formed directly in solution as a result of the interaction of the diamagnetic compound dinitrole or dinitronaphthalene with BSA reaction centers on which oxygen molecules were adsorbed. Quantitative electron paramagnetic resonance spectroscopy demonstrated that the number of reaction sites of the BSA molecule increased by a factor of ≃2 as a result of THz exposure. THz irradiation excites definite collective rotational motions, which partially eliminate steric hindrance for the adsorption of molecular oxygen on the functional groups of BSA.

Thus, there are several mechanisms for the response of living cells to THz radiation:

- changes in membrane permeability and the redistribution of electric charge on the cell membrane;
- changes of the ratio of concentrations of bound and free water;
- modulation of intracellular processes caused by excitation of resonant vibrations of macromolecules that make up the cell membrane and cytoskeleton;
- a change in protein conformation associated with the certain collective rotational movements.

This review examines the generalized terahertz range (0.1–100 THz), which includes the rotational and vibrational components of the molecular spectrum. Terahertz waves can significantly enhance the components of molecular motion, such as twisting, stretching and bending, through resonant excitation.

The role of water molecules is also important, which can be considered as a universal marker in the terahertz frequency range, which is sensitive to various vital processes occurring in living tissues and cells.

## Impact of THz radiation on cancer cells and tissues

**Fig. 11:**
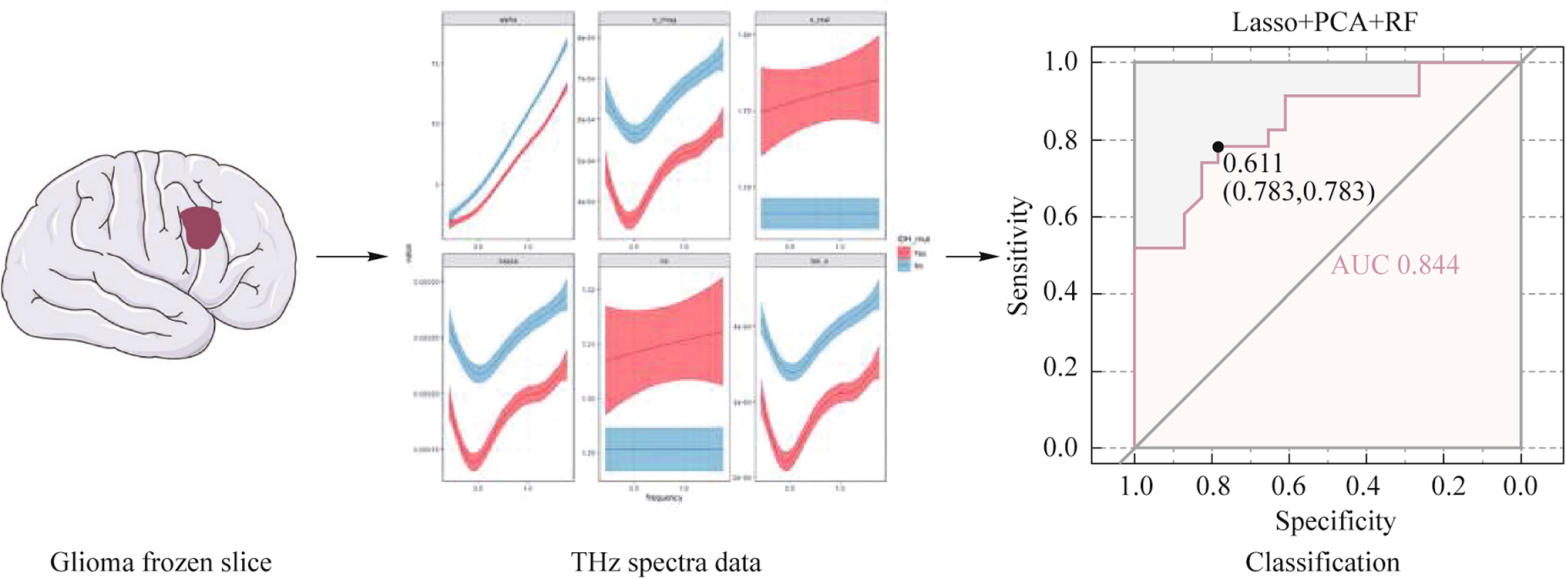
Study of predicting the mutational status of IDH glioma based on THz spectral data using the THz-TDS technique [73]

**Fig. 12:**
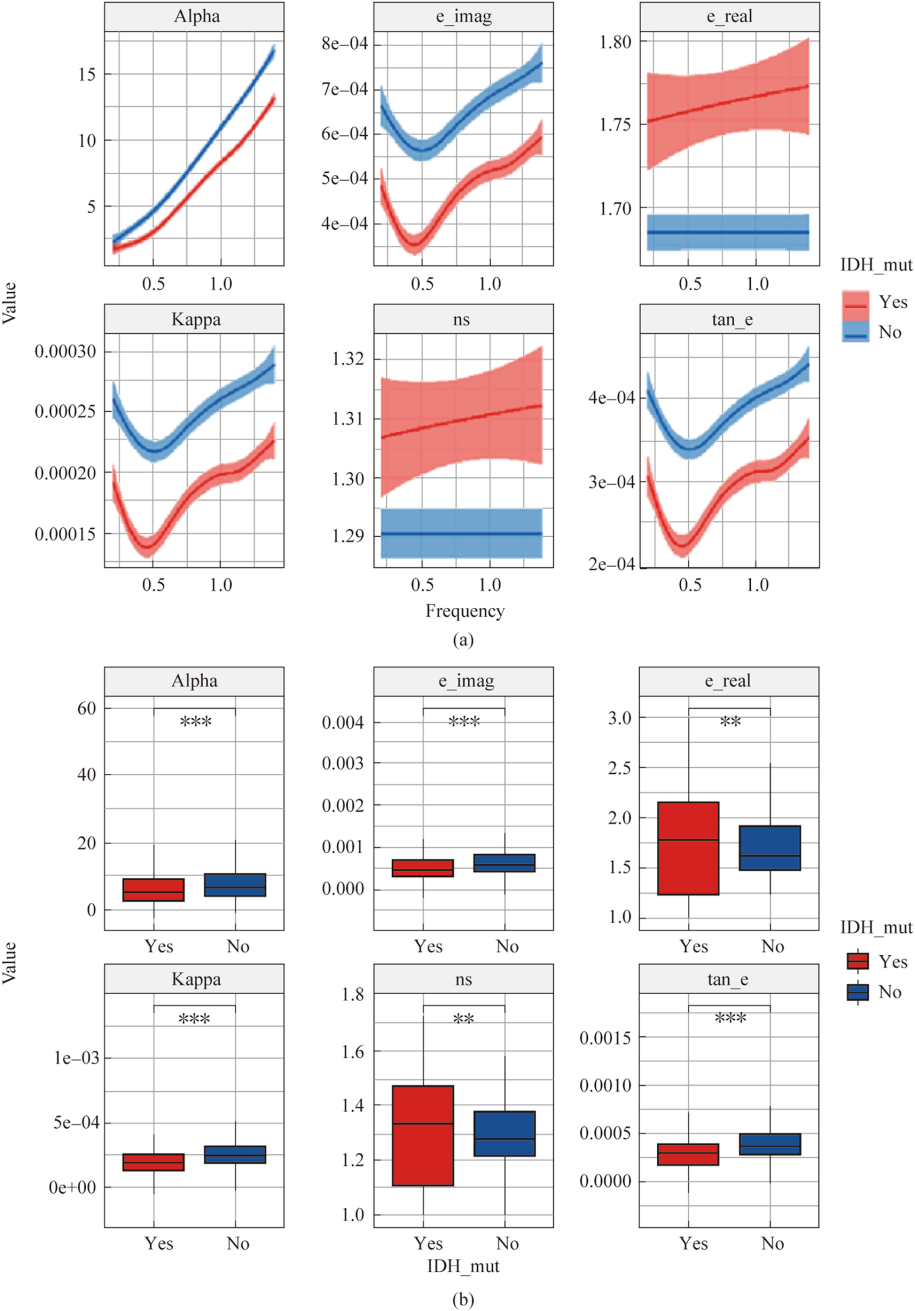
**a** THz spectra for different IDH mutation states. **b** Parameters characterizing the features of IDH mutation. Red and blue colors represent groups of mutants. Alpha = absorption coefficient, ns = refractive index, kappa = extinction coefficient, e_real = dielectric constant, e_imag = dielectric loss factor, and tan_e = dielectric loss tangent (*p* < 0.05, “***” = 0.001, “**” = 0.01, “*” = 0.05) [73]

The emergence of malignant tumors is characterized by the appearance of uncontrollably dividing cells capable of invasion into adjacent tissues and metastasis to distant organs. The development of drugs and methods for treating malignant tumors is an important and still not fully resolved scientific problem. It is believed that 30% to 60% of cases of malignant tumors can be prevented using existing medical technologies [107]. Gliomas are the most common type of primary tumor of the human central nervous system. In [73], a study was carried out to predict the mutational status of isocitrate dehydrogenase (IDH) of glioma based on terahertz spectral data obtained by terahertz time-domain spectroscopy (THz-TDS) (Fig. 11). Mutation status is an important molecular biomarker of diffuse gliomas in adults. These results indicate that gliomas with different IDH mutation status have different spectral characteristics in the THz range, and the use of terahertz spectroscopy can establish a predictive model of IDH mutation status, providing a new way to study glioma. Spectral analysis was used in the range of 0.2–1.4 THz. It has been shown that prediction of the IDH mutational status of glioma can be based on measured values of absorption coefficient, dielectric loss coefficient, extinction coefficient, dielectric loss tangent, dielectric constant and refractive index (Fig. 12).

As shown in Fig. 12b, the IDH mutation status of glioma determines differences in the values of absorption coefficient, dielectric loss, extinction and dielectric loss tangent, which opens the possibility of using these characteristics to monitor such status. In connection with the advent of a non-invasive method for determining the status of IDH mutation based on terahertz spectra, the role of this molecular pathological diagnostic indicator of glioma in the glioma classification system should increase and influence the quality of prognosis and treatment of glioma.

The works [62, 108] carried out a study of the genotoxic effect of high-intensity THz radiation on fibroblasts and tumor cells of the central nervous system. Data were obtained on the impact of high-intensity pulses of THz radiation with a wide spectrum (0.2–3.0 THz) on cell cultures. It was shown that no genotoxic effects of THz radiation were observed in fibroblasts unless the peak power density exceeded 21 GW/cm^2^ (pulse width 95 ± 5 fs at the full-width half-maximum (FWHM)). The reason for this is the strong attenuation of THz radiation in the experimental setup, where only the maximum THz pulse energy of 10 μJ reached the cells under study.

**Fig. 13:**
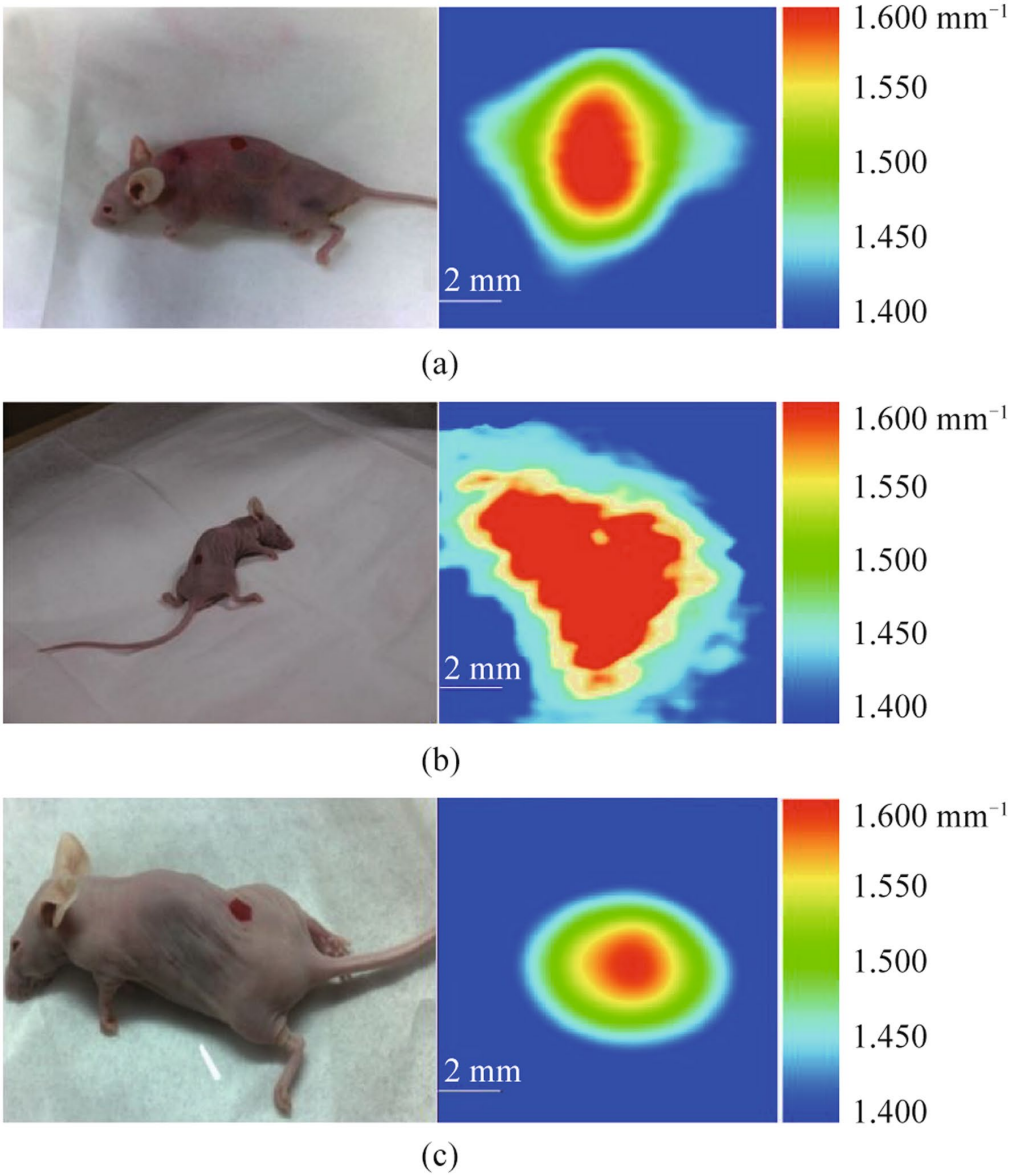
THz images of breast cancer in three mice in the frequency range 108–143 GHz. **a** The projection area of the cancer tumor is about 0.480 mm^2^. **b** The projection area of the cancer tumor is about 0.853 mm^2^. **c** The tumor volume in a mouse is about 0.704 mm^3^ [72]

The pathophysiological features of malignant tumors of the central nervous system determine the appearance of a number of morphological phenomena, such as increased vascularity, edema, and necrosis. These phenomena cause an increase in the water content in the tissues under study and thus open up new possibilities for the application of THz technology in intraoperative neurodiagnostics, including the determination of tumor boundaries. This line of research is quite new and, despite the small volume of accumulated research material, is undoubtedly extremely promising for the creation of new diagnostic approaches [109]. The authors of [72] successfully demonstrated the ability of THz imaging to detect a small breast tumor and distinguish cancerous tissue from fat (Fig. 13). The authors found that the absorption of THz radiation by breast tissue decreases at low frequencies, so a frequency of 108 GHz was chosen for in vivo imaging.

This work proposes a method for the preliminary diagnosis of breast cancer smaller than 1 mm^3^, which is much more effective since the current detection limit of X-ray mammography is 2 mm^3^. Since the quality of prediction of breast cancer development using artificial intelligence (AI) technologies depends on the details of diagnostic data, the use of the THz diagnostic method with higher resolution is likely to bring a new level of development of methods for diagnosing and treating breast cancer. In addition to new THz diagnostic options, the mechanisms of cancer therapy are of interest. Many primary and metastatic tumors have unusual energy metabolism, since aerobic glycolysis is most favored by glucose homeostasis in cancer cells [31].

**Fig. 14:**
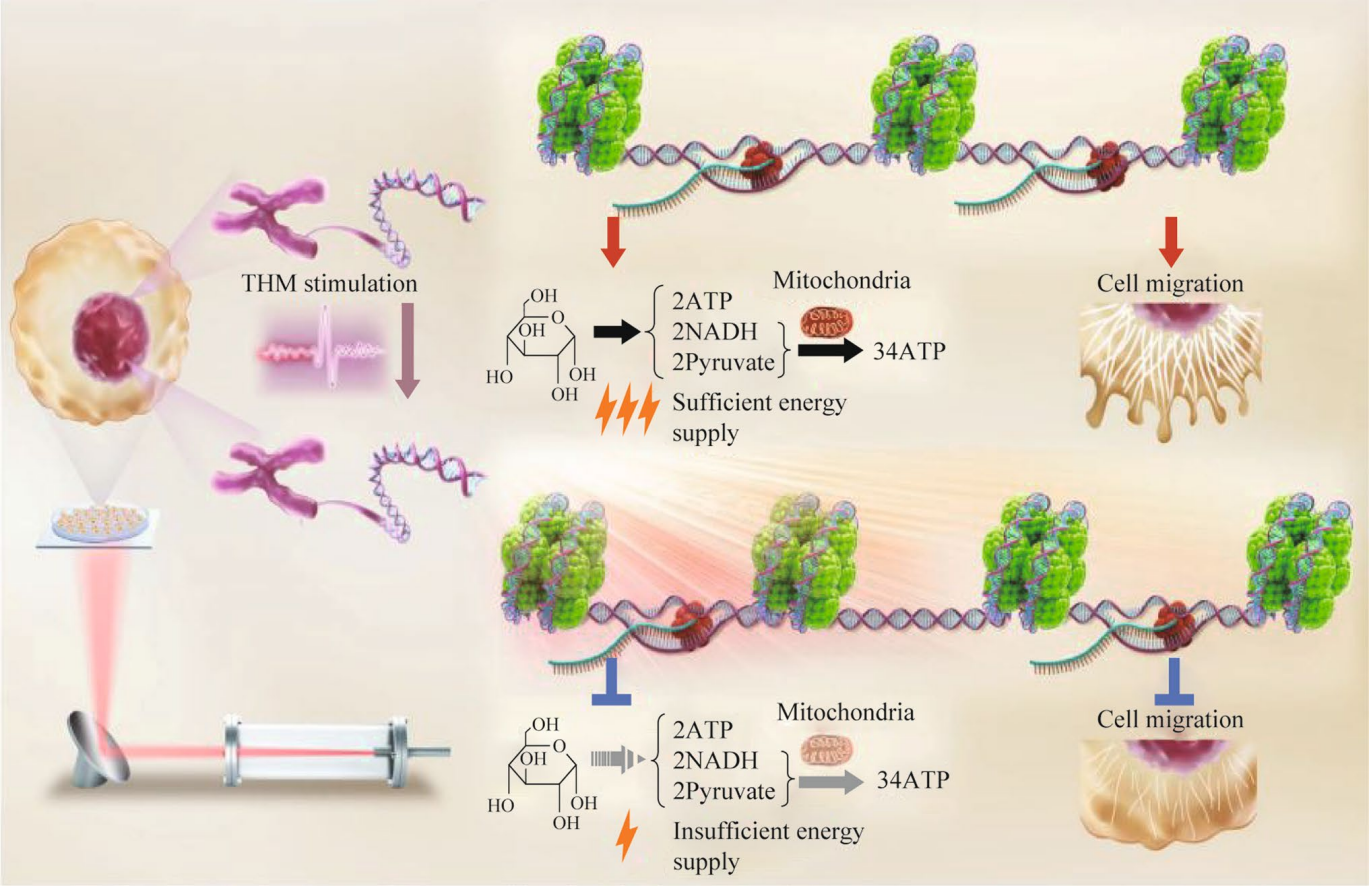
Scheme of the inhibitory effect of THM on cancer cells. THM at a specific wavelength (3.6 µm) significantly inhibited the migration and glycolysis of cancer cells [4]

The use of non-single-frequency generators in the experiment increases the likelihood of successful exposure to radiation. Authors of [4] used a frequency-tuning light source to evaluate the long-term and fundamental biological effects of THz radiation on cancer cells. Different phase matching angles corresponded to different frequencies, ranging from 41.64 to 130.34 THz, and the output power depended on the wavelength. The pump laser was a Q-switched, diode-pumped Nd:YAG electro-optical laser (laser pulse repetition rate was 10 Hz). The maximum pulse energy reached 850 mJ. The pulse duration was approximately 10 ns, and the laser beam divergence angle was < 0.5 mrad. The authors showed that radiation with a wavelength of 3.6 μm (~ 83 THz, *P* = 0.3 mW, maximum electric field amplitude was 2.4 V/nm) significantly increases the binding affinity between DNA and histone (Fig. 14), providing a theoretical possibility of interfering with the functioning of cancer cells due to THz modulation (THM). It was found that low-power 0.3 mW radiation at a frequency of 83 THz can successfully inhibit the migration of cancer cells by 50% and reduce glycolysis by 60%. In the experiment, mice were inoculated with HCT116-Luc cells (3 × 10^6^ cells per mouse) via intrasplenic injection pre-stimulated with THM of 3.6 μm-radiation for 30 min (THM group, *n* = 7) or untreated cells (control group, *n* = 7).

It is known that compared to normal cells, cancer cells have significantly increased metabolic demands. To meet the need for more energy, cancer cells must consume additional glucose and redirect nutrients into macromolecular synthesis pathways to produce lactic acid and support their proliferation and metastasis. When the expression of metabolism-related genes, which play a critical role in cell growth and survival, changes dramatically, glycogen metabolism in cancer cells is altered [4]. Low-intensity THM stimulation at a wavelength of 3.6 μm can lead to dramatic changes in gene expression that inhibit glucose metabolism, hypoxia signaling, and ultimately the migratory ability of cancer cells.

Thus, THz radiation has a diverse effect on living cells, including cancer cells, which manifests itself in a violation of their structure and, accordingly, functions, namely the permeability of cell membranes, and, ultimately, cell viability. The pathophysiological features of malignant tumors are characterized by a high degree of hydration compared to normal tissues. Due to the participation of hydrogen bonds in the absorption of THz waves by a biological medium with high water content, these waves are considered a promising noninvasive tool for the diagnosis and therapy of cancer.

## Conclusion

The presented data show that THz radiation has a variety of effects on cells, which are manifested in the disruption of the properties of cell membranes, the formation of pores, the activation of ion channels, and changes in their proliferation and viability [110]. Possible mechanisms that determine the reaction of cells to THz radiation may be the following:

-  a change in the conformation of membrane proteins, which triggers an intracellular cascade of regulators of the genetic and enzymatic systems and the permeability of cell membranes for various substances;
-  a change in the conformation of membrane proteins that perceive external regulatory signals;
- change in the conformation of membrane proteins that are pumps or channels for the transport of various substances into and out of the cell;
-  redistribution of the electric charge on the cell membrane;
-  excitation of resonant oscillations of macromolecules that make up the cell membrane and the cytoskeleton as a whole.

Thus, fundamentally, THz radiation does not cause the breaking or restoration of chemical covalent bonds, since the quantum energy is insufficient for this, 1 THz − 4.1 meV. However, this radiation, in its frequencies, falls into the region of vibrational–rotational movements of biological molecules and water and can excite energy levels of vibrational–rotational transitions of proteins and water, and thereby change the spatial conformation of proteins, which can affect various interactions between proteins, protein and water molecules.

It is generally accepted that there are several mechanisms that determine the effect of the response of living cells to THz radiation, in particular [1]:

-  redistribution of electrical charge on the cell membrane, changing the ratio of concentrations of bound and free water;
-  excitation of resonant vibrations of macromolecules that make up the cell membrane and the cytoskeleton as a whole;
-  change in the conformation of membrane proteins.

Water molecules themselves can be considered as a universal marker in the THz frequency range, which is sensitive to various vital processes occurring in living tissues and cells. Compared to what is traditionally described in dielectric spectroscopy, in the THz frequency range water as a marker allows one to obtain new information about biological systems. Moving from the gigahertz (GHz) to the THz range, we are gradually approaching various vibrational–rotational processes that are determined by the interaction of water molecules with surrounding molecular systems [23]. Biomacromolecules, being excited, absorb part or all of the energy of electromagnetic waves, depending on the frequency of the incident radiation [26]. Since the generalized terahertz range (0.1–100 THz) partially overlaps with the vibration spectrum of biomolecules, terahertz waves can greatly enhance vibrations of biomolecule bonds such as twisting, stretching, and bending through resonant excitation [111]. However, early studies of the biological effects induced by optical stimulation focused on the infrared region, which promotes the strong absorption of incoming energy by water and its conversion into heat [112, 113]. While heat alters transmembrane capacitance or ion channel activity and hence induces biological responses, it inevitably also causes a transient increase in local temperature. On the other hand, terahertz wave modulation is seen as a promising approach for interfering with biophysical processes without being damaged by electromagnetic radiation. The study of non-thermal biological effects of infrared radiation has attracted close attention from both opticians and biologists. In addition, THz waves with low photon energy are unlikely to cause ionizing effects, thus will not damage genome integrity as other radiation intervention approaches might [112].

It can be concluded that currently there is no full consensus in the scientific community as to whether THz radiation has a damaging effect on biological objects at various levels of organization [83, 114]. Therefore, an increase in studies using THz radiation to monitor the activity of uncontrolled dividing cells is expected in the near future. The development of new high-resolution THz diagnostic methods in combination with AI technologies will take cancer diagnosis and therapy to a new level. It is obvious that more and more new data will appear soon for THz diagnostics and therapy of tumor oncological processes. In addition, theranostics technologies, where THz radiation from the same source is used first for diagnosis and then at increased energy parameters for therapy within a single protocol, have not yet received proper development, but are undoubtedly promising.

## Data Availability

The data that support the findings of this study are available from the corresponding author, upon reasonable request.

## Abbreviations

AI: Artificial intelligence
BSA: Bovine serum albumin
CD: Circular dichroism
DD: Double Debye
DO: Double damped oscillator
EMR: Electromagnetic radiation
HFTS: High frequency terahertz stimulation
FP: Fluorescence polarization
GFP: Green fluorescent protein
ICNIRP: International Commission on Non-Ionizing Radiation Protection
IDH: Isocitrate dehydrogenase
MSC: Mouse stem cells
OCA: Optical clearing agent
PML: Perfect matching layer
RNA: Ribonucleic acid
THM: Terahertz modulation
THz: Terahertz
THz-TDS: Terahertz time-domain spectroscopy
UV: Ultraviolet
WHO: World Health Organization
